# Boosting microfluidic microbial fuel cells performance via investigating electron transfer mechanisms, metal-based electrodes, and magnetic field effect

**DOI:** 10.1038/s41598-022-11472-6

**Published:** 2022-05-06

**Authors:** Mohammad Shirkosh, Yousef Hojjat, Mohammad Mahdi Mardanpour

**Affiliations:** 1 Department of Mechanical Engineering, Tarbiat Modares University, Tehran, Iran; 2 Department of Bioengineering, McGill University, Montreal, QC Canada

**Keywords:** Fuel cells, Biofuels

## Abstract

The presented paper fundamentally investigates the influence of different electron transfer mechanisms, various metal-based electrodes, and a static magnetic field on the overall performance of microfluidic microbial fuel cells (MFCs) for the first time to improve the generated bioelectricity. To do so, as the anode of microfluidic MFCs, zinc, aluminum, tin, copper, and nickel were thoroughly investigated. Two types of bacteria, *Escherichia coli* and *Shewanella oneidensis MR-1*, were used as biocatalysts to compare the different electron transfer mechanisms. Interaction between the anode and microorganisms was assessed. Finally, the potential of applying a static magnetic field to maximize the generated power was evaluated. For zinc anode, the maximum open circuit potential, current density, and power density of 1.39 V, 138,181 mA m^-2^ and 35,294 mW m^-2^ were obtained, respectively. The produced current density is at least 445% better than the values obtained in previously published studies so far. The microfluidic MFCs were successfully used to power ultraviolet light-emitting diodes (UV-LEDs) for medical and clinical applications to elucidate their application as micro-sized power generators for implantable medical devices.

## Introduction

Microbial Fuel Cells (MFCs) are promising green and renewable bioelectricity generation approaches that utilize microorganisms as biocatalysts to harvest energy from organic substrates or biomass^1^. Besides, the numerous applications of MFCs in wastewater treatment and biosensing^2^, powering microbial electrolysis cell (MEC) for biohydrogen production^3^ and beyond that in point of care diagnostics devices^4^ attract academic attention. The latter applications have come to fruition with the aid of microfluidic technology that offers unique advantages of integrating an entire cell on a chip.

Being able to power miniaturized systems for portable, wearable^5^ and implantable medical devices (IMDs)^6^, having shorter response time, controlling operating parameters precisely^1^, and finally achieving a better understanding of biofilm formation and biological interactions^7^ are marked as top features of microfluidic MFCs. However, practical applications of microfluidic MFCs are still limited due to low output power density and high fabrication cost.

The generated power depends on a variety of factors, including physical (electrode materials, membrane and cell configuration), biological (microorganism and substrate type and electron transfer mechanisms in microorganisms) and operating (temperature, pH, external resistance and flow rate) parameters^8–10^. As direct or indirect interaction between the microorganism and the anode surface takes place to transfer extracellular electrons, the anode electrode plays a critical role in this process. Besides, the impact of anode structure and materials, particularly their biocompatibility, porosity, topography, roughness and potential, could remarkably affect biofilm formation and internal resistance of microfluidic MFCs^11^. A promising alternative anode will significantly increase the output power density of microfluidic MFCs to accelerate the transition of this technology from fundamental research to commercial applications.

Since the inception of MFCs technology, numerous studies have been conducted to find an anode electrode with all mentioned characteristics. Regardless of carbon-based electrodes investigation^12^, over fourteen metal-based electrodes, such as nickel (Ni), gold (Au), copper (Cu), molybdenum (Mo), zinc (Zn), tin (Sn), and aluminum (Al), are used as anode electrodes in MFCs^13,14^. The results indicated that Mo has a higher current density than other metals and carbon-based electrodes. This was the initial step toward introducing metal-based electrodes as a competitive alternative to carbon-based electrodes. Moreover, several modification strategies, such as the incorporation of nickel nanostructures^15^ and surface modification with Fe_3_O_4_ nanospheres and reduced graphene oxide^16^, have been developed to improve biocompatibility, surface area to volume ratio and electrical conductivity^17^.

An intriguing result was observed during the use of metal-based electrodes, indicating that they are potentially more promising anode materials than carbon-based electrodes. A three-fold increase in power density has been reported for an Sn-coated copper mesh over a graphite electrode^18^. Metal-based electrodes are mechanically stronger, have higher electrical conductivity, and are more cost-effective. Except for Ni^19^ and Au^20^, no other metal-based electrode has been thoroughly investigated in microfluidic MFCs to date. The interaction of microorganisms and electrode type is a critical issue that has not been considered fastidiously.

Exoelectrogenic microorganisms act as biocatalysts for MFCs to degrade organic substrates while simultaneously generating extracellular electrons. Electrons can be transferred directly from bacteria to electrodes, such as nanowires (produced by species such as *S. oneidensis MR-1*), or via self-produced mediators such as cytochrome c generated by *E. coli*^9^.

The interpretation of electron transfer mechanisms in a mixed culture composed of a deluge of bacteria is not yet fully understood. For instance, copper as the anode electrode generated current densities of 15^13^ and 0 Am^-214^ using two distinct *Geobacter* dominant mixed cultures. On the other hand, investigation of microfluidic MFCs inoculated with pure cultures such as *S. oneidensis MR-1*^21^, *E. coli*^19^ and *Geobacter*^22^ resulted in a straightforward contemplation. However, no report is evaluating and comparing electricity generation under identical operating conditions using electrode types and electron transfer mechanisms.

Among the numerous strategies used to intensify bioelectricity generation in previously published studies, four techniques are worth noting. First, adding chemically synthesized mediators (e.g. methylene blue) to facilitate the transfer of produced electrons^23^; second, knocking out interfering genes in the intracellular metabolic process of bioelectricity production^24^; third, promoting mutant strains to enhance bacterial attachment to the anode and increase current density by more than 50%^25^; and fourth, enhancing biofilm formation by combining the effective genes of E. coli and *S. oneidensis MR-1*. The final technique increased the produced power density by 2.8 fold (from 61 to 167.6 mW m^-2^)^26^. Due to the high cost of exogenous mediators and their potential toxicity to microorganisms, commercialization of enriched microfluidic MFCs using this technique has cast doubt on its commercialization^9^. Besides, metabolic engineering of exoelectrogenic microorganisms is an expensive and time-consuming process that stimulates academic research into alternative techniques for enhancing bioelectricity.

Along with the potential of metal-based electrodes and the utilization of electron transfer mechanisms, applying a static magnetic field is another viable method for improving bioelectricity generation. It has been reported that permanent magnets coupled to the anode^27^ or cathode^28^ of MFCs enhance bioelectricity generation. This phenomenon was first introduced by Moore, attributing an improvement in the metabolism of electrogenesis microorganisms^29^. Furthermore, it has been indicated that coupling an electrode with permanent magnets accelerates electron transfer between microorganisms and electrodes^30^. The effects of applying a magnetic field include fast start-up and internal resistance (about a 39% decrease) of the MFCs, which finally enhanced the produced power density by more than 31%. However, an excessive increase in magnetic field intensity may negatively affect power generation^27^. It is self-evident that there is an optimal magnetic field intensity for maximizing bioelectricity production. The effect of magnetic fields on the performance of microfluidic MFCs as a platform for improving power generation should be thoroughly investigated.

Regardless of how meticulously metabolic processes of microorganisms are investigated or how genetic engineering is used to improve electron transfer, a broad perspective on enhancing the interaction between microbes and electron acceptors as a cost-effective and straightforward method should be taken into account. The aims of this study are, first, to assess metal-based electrodes as the anodes of microfluidic MFCs and improve bioelectricity production. Second, investigating the effect of electron transfer mechanisms and the interaction of the anode electrode with microorganisms on the overall performance of the system. Finally, assessing the potential application of a static magnetic field to maximize the generated power. Since the final applications of the manufactured microfluidic MFCs can be oriented to medical devices, the utilization of non-pathogenic bacteria is crucial. Two non-pathogenic species, *S. oneidensis MR-1* with direct electron transfer and *E. coli* with indirect electron transfer, were inoculated in the microfluidic MFCs under the same conditions. Besides, the capability of microfluidic MFCs to power ultraviolet light-emitting diodes (UV-LEDs) for medical and clinical applications was evaluated for the first time.

## Materials and methods

### Assembly of the microfluidic MFCs

**Figure 1:**
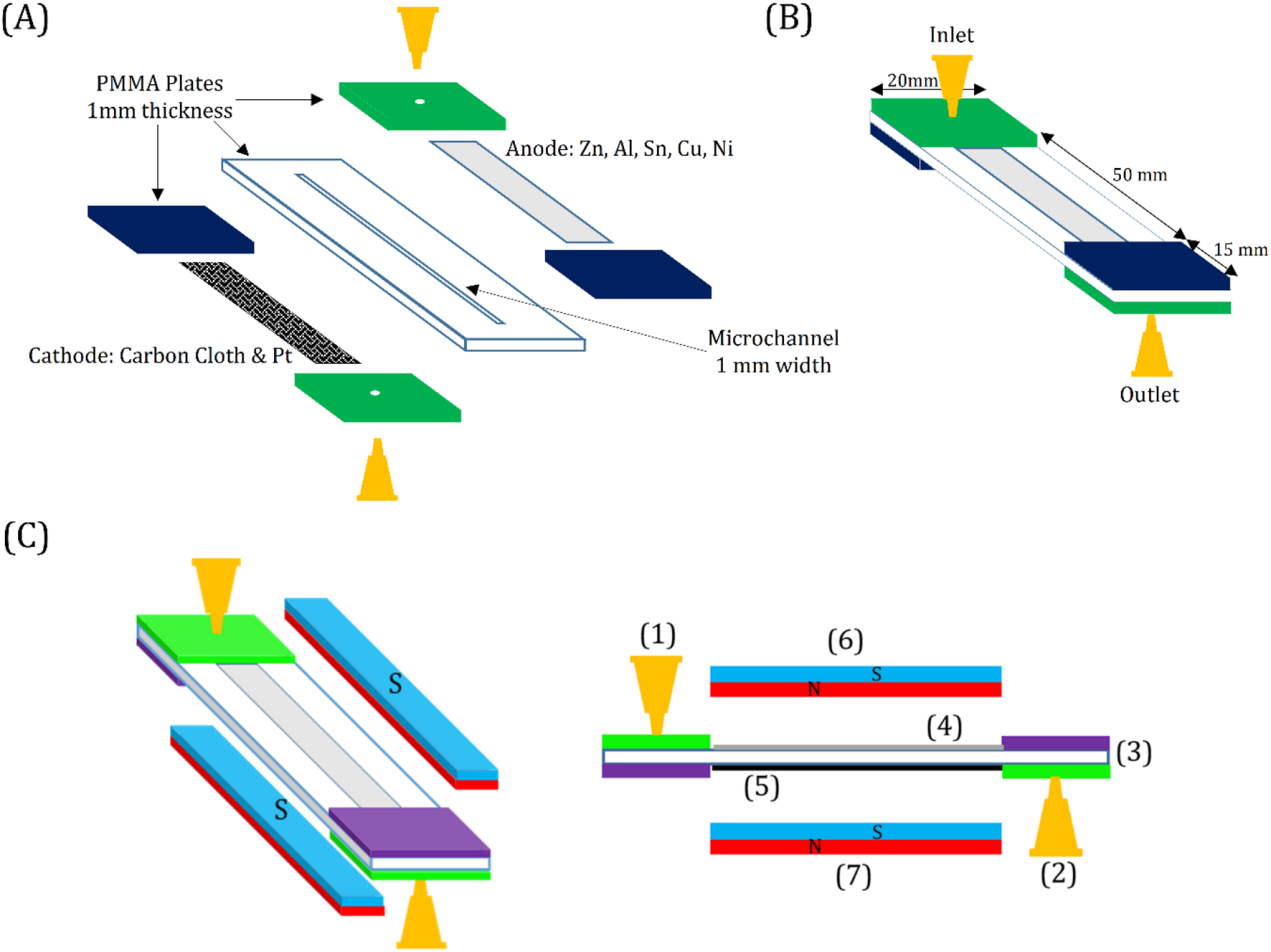
Schematic illustration of the microfluidic MFC: (**A**) exploded diagram and (**B**) fabricated device. (**C**) Schematic details of applying a static magnetic field by Nd–Fe–B permanent magnets. (1): substrate inlet; (2): substrate outlet; (3) microchannel and cell body; (4): anode; (5): cathode; (6): upper magnet; (7): lower magnet.

Figure 1A depicts a schematic exploded view illustration of the proposed microfluidic MFC. A rectangle with a width of 1 mm and a length of 65 mm was cut on a polymethyl methacrylate (PMMA) plate with a thickness of 1 mm (Cho Chen, Taiwan) by a laser beam and then completely removed to form a straight microchannel on the plate that serves as the anolyte compartment of the microfluidic MFCs. So, the height of the microchannel was equal to the thickness of the PMMA plate. A straight microchannel was chosen over a spiral channel^3^ or square geometry^15^ due to its superior performance. Two holes for each cell with a diameter of 1.6 mm were also cut on two PMMA plates, and syringe tips (16G, Changzhou Shuangma Medical Devices, China) were inserted into the plates to provide an inlet and outlet for substrate injection and effluent removal. The laser cutting process was conducted using a CO_2_ laser machine (Model CMA1390-LG, GD Han’s Yueming Laser, China) with power, stand-off distance, and speed of 50 W, 6.5 mm, and 70 mm s^-1^, respectively. The fabricated PMMA plates were attached by chloroform glue to make the main body of the device. Then, as shown in Fig. 1B, the prepared anode was placed on one side of the device, and the cathode was placed on the opposite side (i.e., in front of the anode). It should be noted that one side of the cathode was exposed to the air. Therefore, the oxygen of the air played a role as an electron acceptor and proceeded cathodic redox reaction. To attach the anode and cathode, epoxy adhesive glue was used. Finally, copper wires with a diameter of 0.5 mm were connected to the corner of the electrodes by epoxy adhesive glue to establish the electrical contacts. The fabricated cell was a single chamber microfluidic MFC containing a 50 µl anolyte compartment with a projected surface area of 0.5 cm^2^.

### Air cathode preparation

Carbon cloth (3 mm × 50 mm) was used as the base of the cathode electrode. On the solution side of the cathode, a catalyst layer was also coated to improve the performance of the microfluidic MFC. In brief, a mixture of platinum and carbon powder (10 wt% Pt/C, Sigma-Aldrich) (0.5 mg Pt/cm2 of Carbon Cloth), Nafion solution (5% Nafion solution, Alfa Aesar™) (66.7 µL/mg of Pt), and isopropanol (33.3 µL/mg of Pt) was prepared and coated on the solution side of the cathode as previously reported^31^.

### Metal-based anode preparation

Zn, Al, Sn, Cu and Ni sheets (99% purity, 0.25 mm thickness, Alfa Aesar) were cut to form a rectangle (3 mm × 50 mm) used as the anode of the microfluidic MFCs. The electrodes were soaked with isopropyl ethanol for 3 min to remove organic residue and then washed carefully with distilled water before the cell assembly.

### Applying a static magnetic field

Nd–Fe–B permanent magnets (grade N42, 50 × 10 × 5 mm) were used and located to generate a static magnetic field, as described in Fig. 1C. The effect of an 86 mT magnetic field on the overall performance of the microfluidic MFC was investigated using the selected metal-based electrode and *S. oneidensis MR-1*. It should be noted that even a weak static magnetic field applied to *E. coli* can have a detrimental effect on the viability of bacteria^32^.

### Microbial culture

*Escherichia coli ATCC-11105* and *S. oneidensis MR-1* were procured from the Sharif University of Technology's Biochemical and Bioenvironmental Research Center (BBRC). *Escherichia coli* was cultured for 24 h in nutrient broth (NB) medium (1 g l^-1^ beef extract, 2 g l^-1^ yeast extract, 5 g l^-1^ peptone, and 5 g l^-1^ NaCl) at 37 °C. *Shewanella oneidensis MR-1* was cultured for 48 h in a shaker incubator (100 rpm) at 30 °C in tryptic soy broth (TSB) medium (17 g l^-1^ Tryptone, 3 g l^-1^ soy, 5 g l^-1^ NaCl, 2.5 g l^-1^ K_2_HPO_4_, and 2.5 g l^-1^ glucose). The microbial enrichment process was conducted under open circuit conditions to achieve a uniform and homogenous biofilm^33^. The microfluidic MFCs were started using a syringe pump to inject a mixture of bacteria and substrates (i.e., *E. coli* with NB and *S. oneidensis MR-*1 with TSB) into the fabricated cells.

Given that *E. coli* transfers electrons via self-produced mediators (i.e., cytochrome c), it is evident that continuous injection of both microbe and medium is required to maintain the rate of electron production. As a result, a mixture of *E. coli* and NB was injected into the system during the operation of the microfluidic MFCs. *Shewanella oneidensis MR-1* transfers electrons directly to the anode via attached bacteria-generated nanowires. After 10 h, the injection of *S. oneidensis MR-1* was stopped, and the system was fed only TSB.

### Calculations and analysis

#### Electrochemical analysis

The open circuit potential (OCP), produced power and current densities as the electrochemical characteristics of the systems were assessed. Every minute, the cell potential was recorded using a multimeter datalogger (PROVA-803). External resistances ranging from 10 to 300,000 Ω were used to obtain the polarization and power density curves. Then, using Ohm’s law, the generated power and current were calculated and normalized using the projected surface area of the air diffusion cathode (0.5 cm^2^).

To demonstrate the capability of the microfluidic MFCs to function as both power generators and disinfection devices in medical and clinical applications, the optimized microfluidic MFC was used to power LEDs (red, white, blue, and UV). The capability of the system for the applications mentioned above is demonstrated by its ability to power red, white, blue, and UV LEDs (DGPY-5 mm). Sustainability of the microfluidic MFCs was determined by monitoring the power consumed by these LEDs over time. Additionally, the light intensity of all LEDs was determined using a light meter (LX-103, Lutron). Three UV-LEDs were used to demonstrate disinfection capabilities of the proposed microfluidic MFCs. Since the mid-twentieth century, the potential for UV-LEDs for use in medical sanitation and to kill pathogenic microbes has been recognized^34^.

## Results and discussion

### Open circuit potential (OCP)

**Figure 2:**
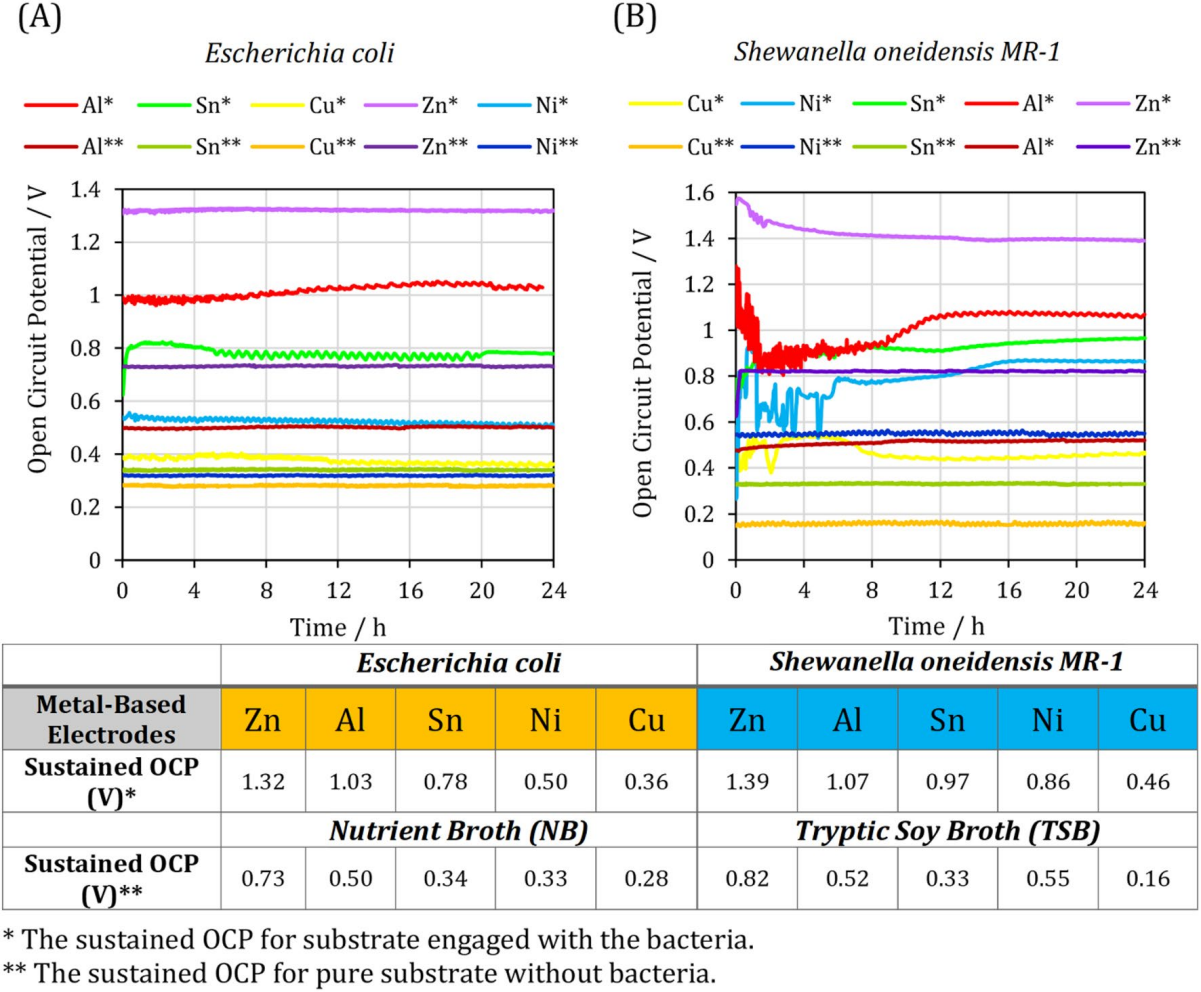
The open circuit potential (OCP) evolution of zinc, aluminum, tin, copper and nickel anode electrodes with (**A**) *Escherichia coli* and (**B**) *Shewanella* oneidensis MR-1 microorganisms and pure substrates.

Open circuit conditions impose the highest external resistance on the cells, resulting in a uniform biofilm morphology and ample time for substrate diffusion into the biofilm^33^. As applied external resistance progressed the oxidation reaction of the organic substrate by biocatalysts to produce significantly more electrons, the biodegradation reaction proceeded with the least amount of driving force possible under open circuit conditions. As a result, the bacteria have sufficient time to form a uniform biofilm. The formed biofilm will be homogeneous, which allows for easier access to the substrate than the heterogeneous biofilm formed under closed circuit conditions. The initial assessment of Zn, Al, Sn, Cu, and Ni was accomplished by tracking the OCP evolution of *E. coli* and *S. oneidensis MR-1* under the same injection condition (0.2 ml h^-1^ flow rate) and inoculation time (Fig. 2).

The OCP evolution of *E. coli* and *S. oneidensis MR-1* in microfluidic MFCs manufactured using various metal-based anode electrodes are depicted in Fig. 2. The sustained OCP values of each cell cultured for *E. coli* and *S. oneidensis MR-1* were arranged by magnitude. Instability in the early stages of OCP evolution in the presence of *S. oneidensis MR-1* (Fig. 2B) could be a result of a variety of factors, including the interaction between substrate and bare anode surface, incomplete biofilm formation, and competition between bacteria to reach the anode surface and form the biofilm^35^.

As illustrated in Fig. 2, the maximum OCPs of 1.32 and 1.39 V were obtained for the Zn anode in microfluidic MFCs cultured with *E. coli* and *S. oneidensis MR-1*, respectively. Zn and Al electrodes exhibit a higher OCP than most carbon-based electrodes in microfluidic MFCs inoculated with *S. oneidensis MR-1*, which could be attributed to the fact that these metals have a higher reduction standard potential and a faster rate of ion transfer in the anolyte than carbon-based electrodes^36^. The Zn anode has the highest OCP, while the Cu anode has the lowest. In Zn-anode microfluidic MFCs, two series of redox reactions could affect the OCP of the cell. First is the redox reaction associated with zinc oxidation, which releases zinc ions into the media as a new electrolyte. The second is the oxidation of organic substrate by bacteria. The incorporation of zinc oxidation could generate a higher potential than the maximum possible theoretical value of 1.14 V^37^, and consequently, the fabricated microfluidic MFC can be considered as a hybrid system. Typical electrochemical reactions of an MFC are oxygen reduction at the cathode and acetate oxidation at the anode^38^. So, possible redox reactions and half-cell potentials are as follows^39,40^:

Microbial fuel cell:

Anode:

1 $$ {\text{CH}}_{3} {\text{COO}}^{ - } + 4{\text{H}}_{2} {\text{O}} \to 2{\text{HCO}}_{3}^{ - } + 9{\text{H}}^{ + } + 8{\text{e}}^{ - } \;\;\;\;\;\;\;\;\;\;\;\;\;\;\; - 0.296 \, V $$

Cathode:

2 $$ {\text{O}}_{2} + 4{\text{H}}^{ + } + 4{\text{e}}^{ - } \to 2{\text{H}}_{2} {\text{O }} \;\;\;\;\;\;\;\;\;\;\;\;\;\;\; + 0.815{\text{V}} $$

3 $$ {\text{O}}_{2} + 2{\text{H}}_{2} {\text{O}} + 4{\text{e}}^{ - } \to 4{\text{OH}}^{ - } { } \;\;\;\;\;\;\;\;\;\;\;\;\;\;\; + 0.401{\text{V}} $$

Furthermore, for a Zn-air battery the redox reactions are mentioned as follows^41^:

Zn-air battery:

Anode:

4 $$ {\text{Zn}} + 4{\text{OH}}^{ - 1} \to {\text{Zn}}\left( {{\text{OH}}} \right)_{4}^{2 - } + 2{\text{e}}^{ - } { } \;\;\;\;\;\;\;\;\;\;\;\;\;\;\; - 1.199{\text{V}} $$

$$ {\text{Zn}}\left( {{\text{OH}}} \right)_{4}^{2 - } \to {\text{ZnO}} + {\text{H}}_{2} {\text{O}} + 2{\text{OH}}^{ - } { } $$

Cathode:

5 $$ \frac{1}{2}{\text{O}}_{2} + {\text{H}}_{2} {\text{O}} + 2{\text{e}}^{ - } \to 2{\text{OH}}^{ - } { } \;\;\;\;\;\;\;\;\;\;\;\;\;\;\; + 0.401{\text{V}} $$

Consequently, the cathode reactions of both systems could be the same and theoretical values of 1.111 and 1.6 V for microbial fuel cells and zinc-air batteries could be achieved, respectively. The OCPs of the microfluidic MFCs with zinc anode were obtained 1.32 and 1.39 V. Regarding the operation of the cells as a hybrid system, the high value of OCP can be attributed to the incidence of above reactions. Even compared to dual-chamber microfluidic MFCs, the sustained OCP of the Zn-anode microfluidic MFC was greater than the obtained OCPs in the previously published studies^21^. The Zn anode demonstrates the first promising step toward commercializing microfluidic MFCs.

A more in-depth examination also reveals the role of the remarkable interaction between biocatalysts and the electrode. The embedded table in Fig. 2 compares the sustained OCP of microfluidic MFCs in the presence and absence of bacteria. For Zn anode, the difference in voltage between the bacteria-inoculated and the medium-only cell was much higher than other electrodes, which reveals the significant role of bacteria in facilitating redox reactions. To investigate the share of each issue in overall power generation, the microfluidic MFC was operated with and without bacteria inoculation, and the maximum power and current densities were obtained about 166.4 mW m^-2^ and 4400 mA m^-2^ without bacteria presence and 14,592 mW m^-2^ and 118,000 mA m^-2^ with bacteria inoculation, respectively. The achieved values indicated that the share of the redox reaction associated with zinc oxidation in overall current and power densities could be estimated about 1.14% and 3.72%, respectively. This demonstrates the critical role of bacteria in organic substrate oxidation and the negligible effect of zinc anode oxidation on the generated bioelectricity. In addition, the internal resistance of the system (as an indicator of anolyte conductivity) with the presence of bacteria was 50 Ω while it was 2000 Ω without bacteria inoculation in the microfabricated cells. This 40 times difference reveals that the overall performance of microfluidic MFC with zinc anode was mainly dependent on the activity of the microorganisms.

### The assessment of the microfluidic MFCs’ performance by power density curves

Concerning the critical role of the anode electrode in lowering the activation overpotential and thereby facilitating redox reactions, the performance of each metal-based electrode can be evaluated. Moreover, the effectiveness of each electron transfer mechanism will be demonstrated.

#### Power density curves

**Figure 3:**
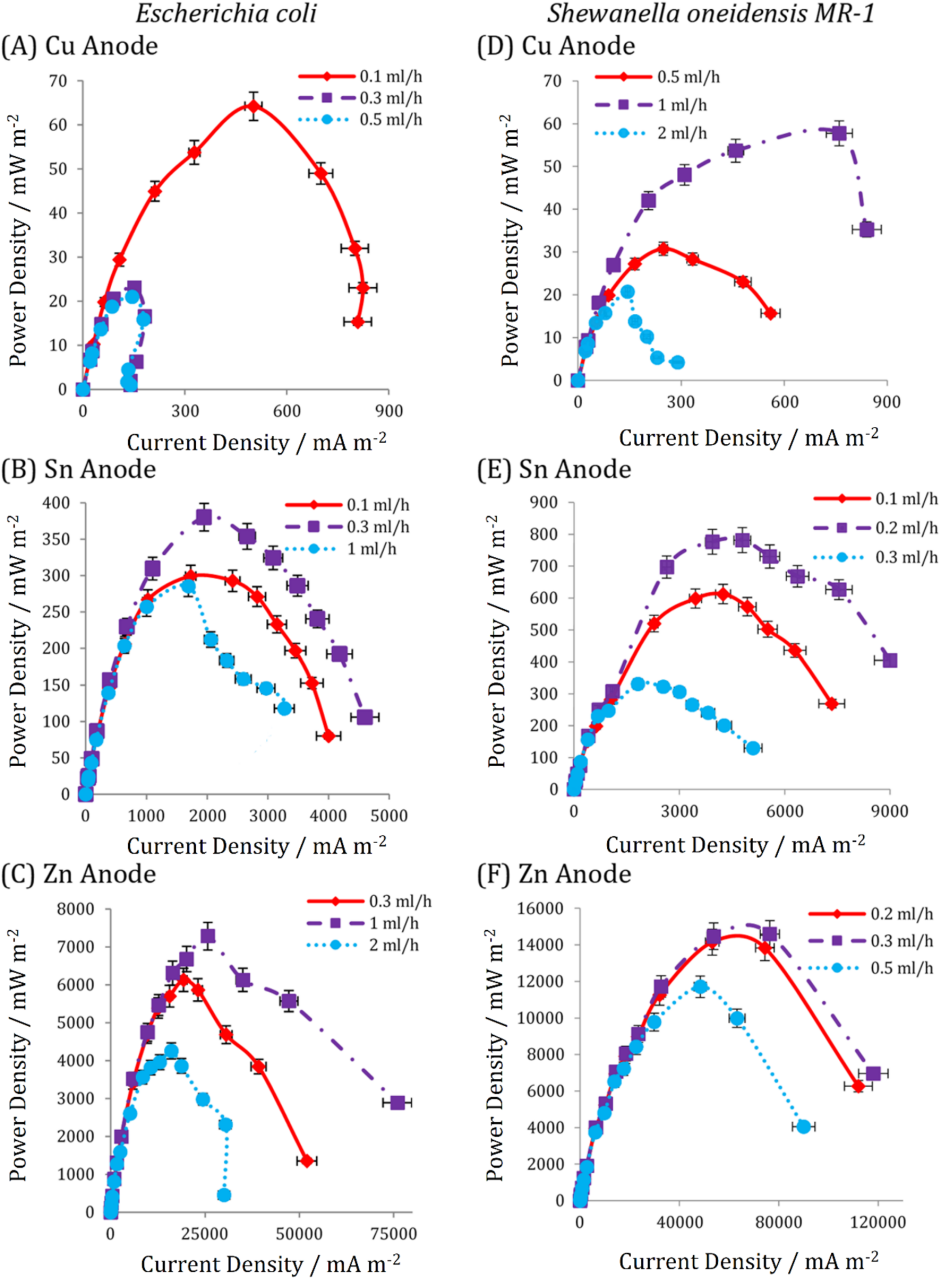
The power density curves for the microfluidic MFCs with different anode electrodes engaged with *Escherichia coli* ((**A**) Cu, (**B**) Sn and (**C**) Zn) and *Shewanella oneidensis MR-1* ((**D**) Cu, (**E**) Sn and (**F**) Zn) under different substrate injection rates. The error bars represent the variation of power and current densities among repeated experiments.

The power density curves of metal-based electrodes (including Cu, Sn and Zn) for *E. coli* and *S. oneidensis MR-1* based on different substrate flow rates are shown in Fig. 3. A detailed explanation of other metal-based electrodes is presented in Supplementary file. Microfluidic MFCs with a Cu anode exhibit the lowest power and current densities, whereas those with a Zn anode exhibit the highest level of these values. Microfluidic MFCs inoculated with *S. oneidensis MR-1* perform better than those inoculated with *E. coli* for Zn, Al, Sn and Ni anodes. Apart from their compatibility with metal-based electrodes, direct electron transfer mechanisms have a lower loss than indirect electron transfer via mobile electron shuttles.

Compared to other metal-based electrodes and other MFCs previously published in the literature (including macro and micro-scale MFCs)^14,22^, the Zn-anode MFC engaged with *S. oneidensis MR-1* produced the highest power and current densities reported to date (about 14,592 mW m^-2^ and 118,000 mA m^-2^, respectively). These results demonstrate a successful match between direct electron transfer mechanisms and the potential of zinc as an anode in a microfluidic MFC. Even for Al and Sn of the microfluidic MFC inoculated with *S. oneidensis MR-1*, the produced power densities are much higher than the carbon-based electrodes^42^, which indicates successful compatibility of nanowires and metal-based electrodes.

The Sn-anode microfluidic MFCs had power densities of 380 and 781.4 mW m^-2^, respectively, when *E. coli* and *S. oneidensis MR-1* were used (Fig. 3B and E). Sn exhibits a power density of 271 and 242 mW m^-2^ when used as the anode in an MFC inoculated with *Geobacter* dominant mix cultures^14,18^. The power density of Sn obtained by *S. oneidensis MR-1* is greater than 2.8 times that reported for *Geobacter* dominant mix cultures, indicating that *S. oneidensis MR-1* has superior biocompatibility and electron transfer to metal-based electron acceptors. Additionally, the benefits of microfluidic MFCs, which enhance bioelectricity generation, should not be overlooked.

As with the OCP results, the microfluidic MFCs with the Cu anode generated lower power and current densities than those with other metal-based electrodes (about 57.76 and 64.25 mW m^-2^, respectively). Anti-biofilm and anti-bacterial properties of Cu^43^ preclude the formation of an effective electron producer layer, which may be the primary reason for the low level of power generation. The performance of Cu as an anode in MFCs has been demonstrated to be a sensitive electron acceptor for the biocatalyst. Maximum power densities of 2 and 69 mW m^-2^ were obtained in Cu-anode MFCs when *Geobacter* dominant culture^13^ and *S. oneidensis MR-1* were used^44^, respectively. This poor performance has been attributed to toxic ion release and corrosion^45^.

The optimal flow rate in microfluidic MFCs is determined by mass transfer conditions^46^, nutrient procurement for biofilm growth^7,47,48^, hydrodynamic stability^49^, and bacteria detachment by avoiding excessive shear stress near the anode surface^7,46^. Regarding the similarity of the cells' geometry in this study to the fabricated microsized-MFC in the work of Mardanpour and Yaghmaei^19^, the range of the substrate flow rate was determined based on the reported values of the mentioned study. The flow rates should be adjusted in the range to establish a continuous flow in the microchannel and inhibit biofilm dehydration on the one hand and prevent biofilm detachment due to the high shear stress on the other hand. The difference among optimal flow rates for the microfluidic MFCs inoculated with different biocatalysts (i.e., *E. coli* and *S. oneidensis MR-1*) indicates a remarkable effect of substrate flow rate on electron transfer mechanisms. Except for Cu, the optimal flow rate for all metal-based electrodes in the microfluidic MFCs utilizing *S. oneidensis MR-1* is lower than that for *E. coli*. Regarding the electron transfer mechanisms for S. oneidensis MR-1 via the attached nanowires on the anode surface, a lower flow rate allows sufficient time for biofilm formation on the electrode and prevents biofilm detachment and substrate flow stress. On the other hand, *E. coli* transfers electrons via self-produced mediators (i.e., cytochrome c) that act as electron shuttles. A higher flow rate can result in a more significant number of shuttles in the microchannel to transfer extracted electrons from the bacteria membrane.

The overshoot phenomenon is another characteristic that can be observed in a power density curve. As external resistance decreases, the amount of current produced and the demand for electrons increases. If bacteria cannot supply the required electrons via redox reactions, an abrupt decrease in current and power occurs, known as the overshoot phenomenon^50^. An increase in substrate flow rate may compensate for decreased current density, provide additional nutrients to microorganisms, and accelerate their metabolic rate in electron production. As a result, overshoot may occur when the flow rate is less than the optimal flow rate for feeding microorganisms. Additionally, at higher flow rates, insufficient time for bacteria and cytochrome-c molecules to reach the electrode surface^19^ may be another factor contributing to the overshoot phenomenon. Considering the power density curves, none of the microfluidic MFCs inoculated with *S. oneidensis MR-1 *experience the overshoot phenomenon. On the contrary, this issue was observed in power density curves of Cu, Ni and Zn inoculated with *E. coli* in flow rates of 0.3, 3 and 2 ml h-1, respectively.

#### Polarization curves

**Figure 4:**
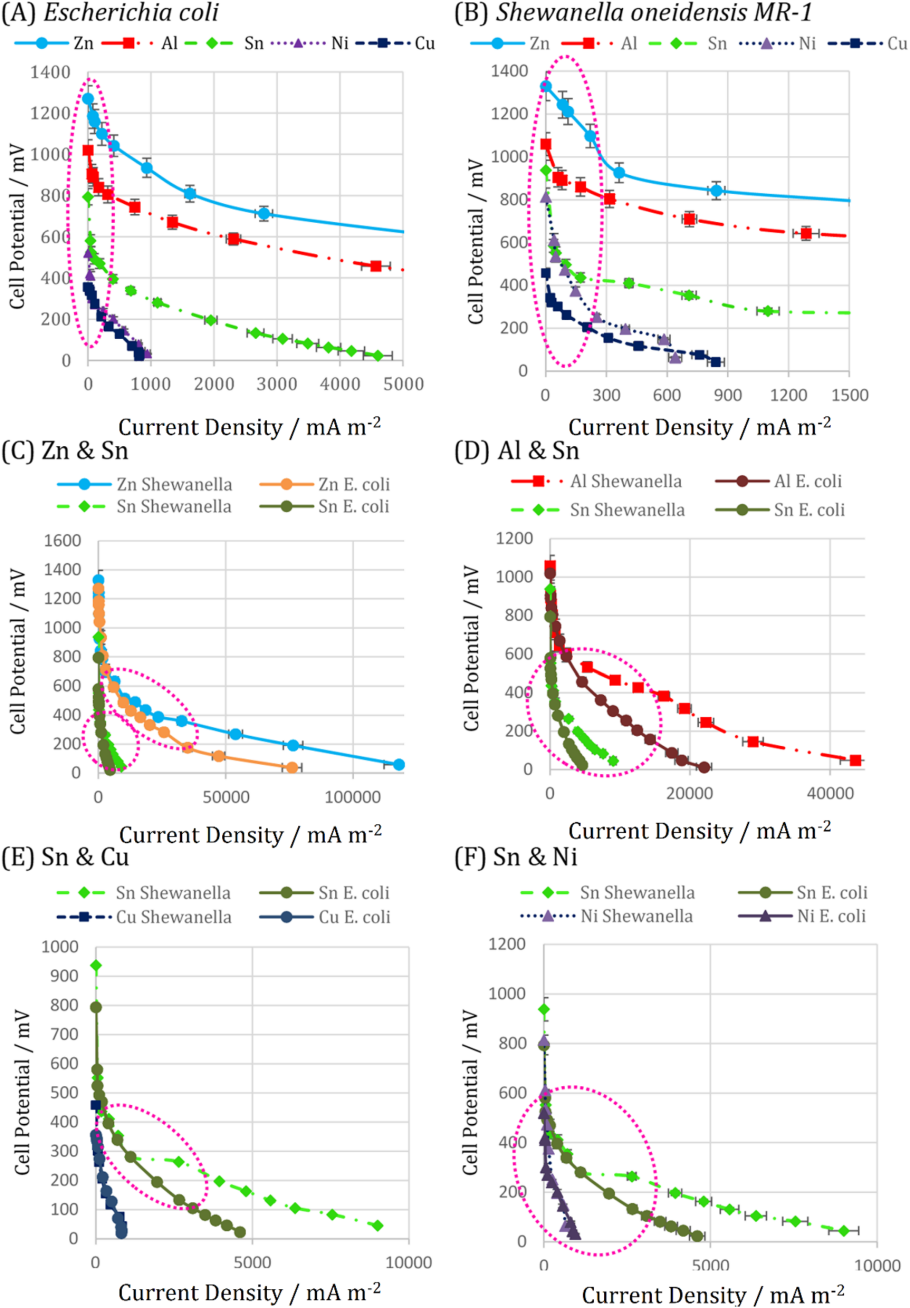
The comparison of activation and ohmic overpotentials of different electrodes and electron transfer mechanisms of the used biocatalysts by polarization curves. (**A**) *Escherichia coli*, (**B**) *Shewanella oneidensis MR-1*, (**C**) Zn & Sn, (**D**) Al & Sn, (**E**) Sn & Cu, (**F**) Sn & Ni. The error bars represent the variation of power and current densities among repeated experiments.

Polarization curves can be used to evaluate the effect of electrode type and electron transfer mechanisms on the overpotentials of the systems. The first, middle, and end parts of a polarization curve can be used to determine the activation, ohmic, and concentration overpotentials, respectively^51^. The polarization curves of various metal-based electrodes for two species with distinct electron transfer mechanisms are shown in Fig. 4. The slope of the initial section of the polarization curve, denoted by the dashed ellipse, indicates the order of activation overpotential. As can be seen, the slope of *S. oneidensis MR-1* polarization curves (Fig. 4B) is less than that of *E. coli-inoculated* microfluidic MFCs (Fig. 4A), which indicates that *S. oneidensis MR-1* requires less activation energy to extract electrons from substrate oxidation than *E. coli*. The metabolic pathways in *S. oneidensis MR-1* may be more streamlined than those in *E. coli*. Furthermore, Zn has less slope than any of those species due to less energy loss during the redox reaction, which may account for lower standard reduction potential of bacteria and increased biocompatibility with Zn anodes. Although Al is a more vigorous reducing species than Zn, the biocompatibility of Zn may have a more significant influence on the performance of microfluidic MFCs. Sn, Ni, and Cu have a greater slope and adjust to their respective positions in the table of standard reduction potentials.

According to the polarization curves in Fig. 4A, no significant difference in the activation overpotential was observed for the Sn, Ni, and Cu anodes of the microfluidic MFCs using *E. coli* as biocatalyst. On the other hand, the significant difference in the slope of the polarization curves in the initial zone of Fig. 4B is visible. When *S. oneidensis MR-1* is inoculated, Sn and Ni exhibit a lower activation overpotential than Cu, which emphasizes the critical role of the anode surface nanowires in reducing activation overpotential.

Regarding the ohmic overpotentials of microfluidic MFCs, which can be determined via the middle zone of the polarization curve, some speculation about the ascendancy of electron transfer mechanisms can be made. The polarization curves of metal-based electrodes used by various microbial species are shown in Fig. 4C–F. The generated power by Sn is higher than Cu and Ni, while it is lower than Zn and Al. This makes Sn an excellent candidate to be used as a reference and compare the electrical characteristics of the other electrodes. So, the polarization curves of each metal-based electrode are shown alongside the Sn polarization curve as a reference to make the comparison perfectly well. As illustrated in Fig. 4C, the slope of the curve in the middle zone (contained within a dashed ellipse) of *S. oneidensis MR-1* is less than *E. coli* in the microfluidic MFC using Zn anode, which is also true for Al and Sn. Because the ohmic overpotential is a measure of the conductivity of the anolyte and the delivery of electrons to electron acceptors, it indicates that nanowires require less energy to deliver electrons than mobile electron shuttles. The exoelectrogenic microbes transferring electrons directly by nanowire (such as *S. oneidensis MR-1*) can be introduced as an alternative to *E. coli* that transfers electrons via self-produced mediators^52^. This substitution in the inoculation of microfluidic MFCs improved the cell performance and its efficiency noticeably. Comparing the results of the previous studies^3,19,53^ revealed the superior performance of *S. oneidensis MR-1* over *E. coli* even when anode surface enriched with nanoparticles to improve the power generation ^15^. In terms of anolyte conductivity, it has been demonstrated that nanowires can transport electrons in a long-range path and consequently increase the biofilm conductivity^54^. So, even a thick biofilm with a network of nanowires may result in a lower internal electrical resistance than mobile electron shuttles. Future research into the calculation of electrical fields in both types of electron transfer processes may prove extremely useful in this area.

The ohmic overpotential of the microfluidic MFCs with Cu and Ni anodes did not show a significant difference (Fig. 4E,F). The difference in electron transfer mechanisms had no discernible effect on the performance of those cells, which may be due to the low proclivity of Cu for biofilm attachment, and the *S. oneidensis MR-1* biofilm cannot play a unique role in electron conduction. Thus, the suspended microbes can contribute primarily to electron transfer, and no significant difference is observed when *S. oneidensis MR-1* or *E. coli* are added to the microfluidic MFC with Cu anode. The scanning electron microscopy images provided additional evidence for the previously mentioned reason for Cu disinclination biofilm formation. In the case of Ni, further research is required to clarify, evaluate, and compare ohmic overpotentials based on electron transfer mechanisms.

#### Effect of a static magnetic field on the generated power

**Figure 5:**
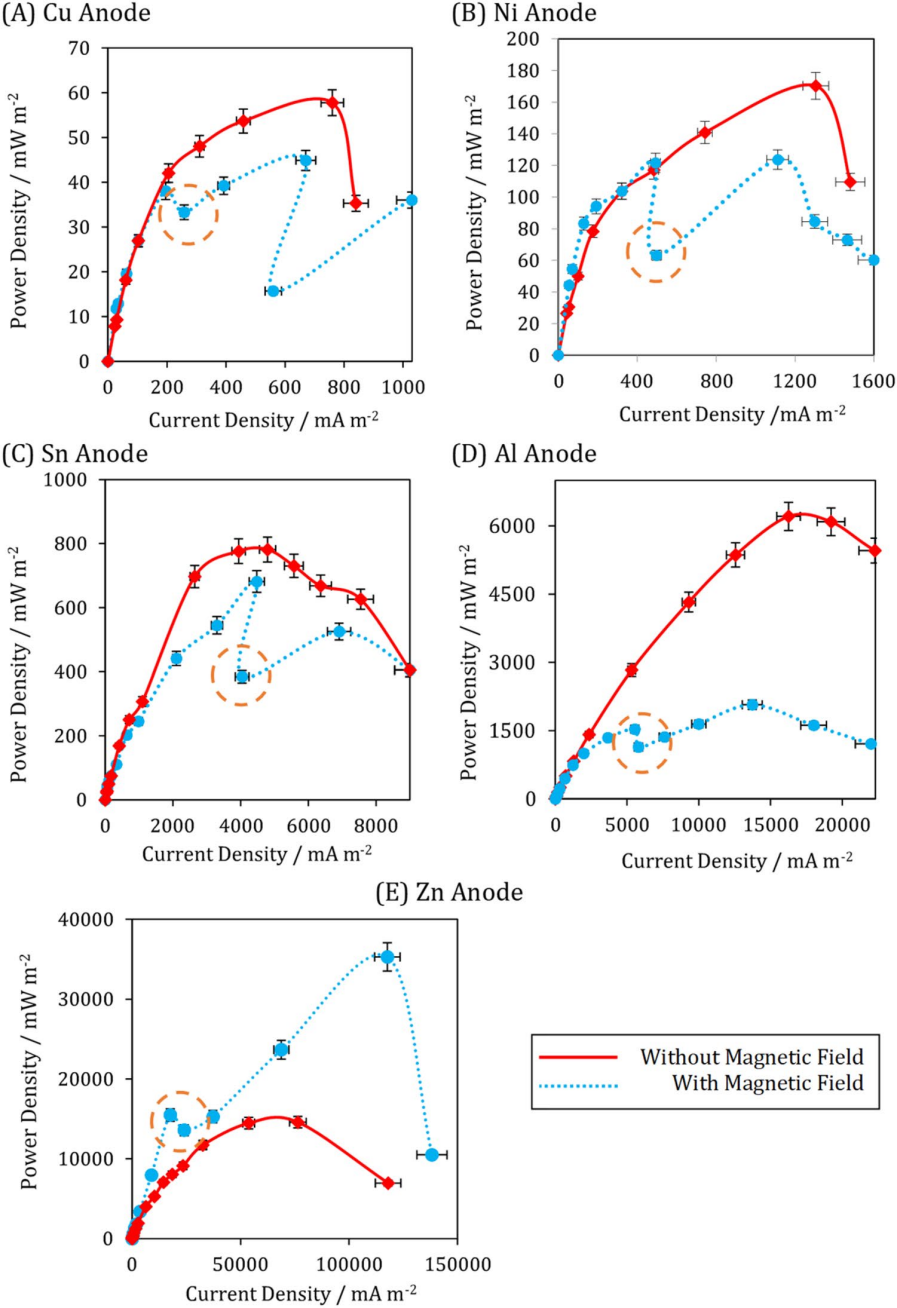
The effect of static magnetic field on the performance of the metal-based electrodes of the microfluidic MFCs engaged with *Shewanella oneidensis MR-1* in the optimized substrate flow rates (Table S1 in Supplementary file). The error bars represent the variation of power and current densities among repeated experiments.

Figure 5 illustrates the effect of applying a static magnetic field of 86 mT on the power density curve of metal-based electrodes. It should be noted that the fabricated cells were operated for almost ten days and continuously exposed to the static magnetic field during this period. As can be seen, except for the Zn-anode microfluidic MFC, the generated power of the other microfluidic MFCs has decreased significantly. In addition, each of them experienced a fluctuation (denoted by a dashed circle). This abrupt decrease is slight for Cu, Al, and Zn anodes, but it is dramatic for Ni and Sn. Applying a static magnetic field reduced the generated power of Cu, Ni, Sn, and Al by 22%, 27%, 13%, and 66%, respectively. For Zn, on the other hand, this magnetic field increased the power density by more than 2.4 times. Although it is difficult to attribute this complex variation to a single parameter, it appears that the magnetic properties of anodes and the metabolic properties of *S. oneidensis MR-1* are more important than other variables.

Regarding the magnetic property of metal-based anodes, Zn and Cu are diamagnetic, Al and Sn are paramagnetic, and Ni is ferromagnetic^55^. The noticeable increase in power density of the Zn anode cannot be attributed solely to its magnetic property, as the Cu anode did not show the same behavior. Furthermore, Al and Sn anodes did not exhibit the same decrease when a static magnetic field was applied. No study has been conducted to examine the variation in metabolic reactions of *S. oneidensis MR-1* when a static magnetic field is applied to the authors' knowledge. Future research will focus on deciphering this subject and the effect of static magnetic field strength on the performance of microfluidic MFCs.

Table S1 in Supplementary file summarizes the major points of the electrochemical analysis discussed in previous figures. When *S. oneidensis MR-1* was used instead of *E. coli*, Zn, Al, Sn, and Ni produced nearly twice the power density, indicating that the direct electron transfer mechanism takes precedence over the mediator-based electron transfer mechanism. Internal resistance values calculated for all electrodes of the microfluidic MFCs almost indicate a decreasing trend in *S. oneidensis MR-1* utilization over *E. coli*. Except for the copper anode, the optimal flow rate in microfluidic MFCs inoculated with *S. oneidensis MR-1* is lower than that in inoculated with *E. coli*. In the case of *S. oneidensis MR-1*, the possibility of bacterial detachment necessitates a decrease in flow rates, whereas the facilitation of electron shuttle in the cells used *E. coli* necessitates an increase in flow rates.

When a static magnetic field is applied, the Zn anode behaves differently than other metal-based anodes. It is not easy to interpret the reasons for the complex variations in the polarization trends of metal-based electrodes. In general, a static magnetic field has a detrimental effect on the power generation of microfluidic MFCs, except for Zn-anode microfluidic MFCs.

### The assessment of the biofilm topology

**Figure 6:**
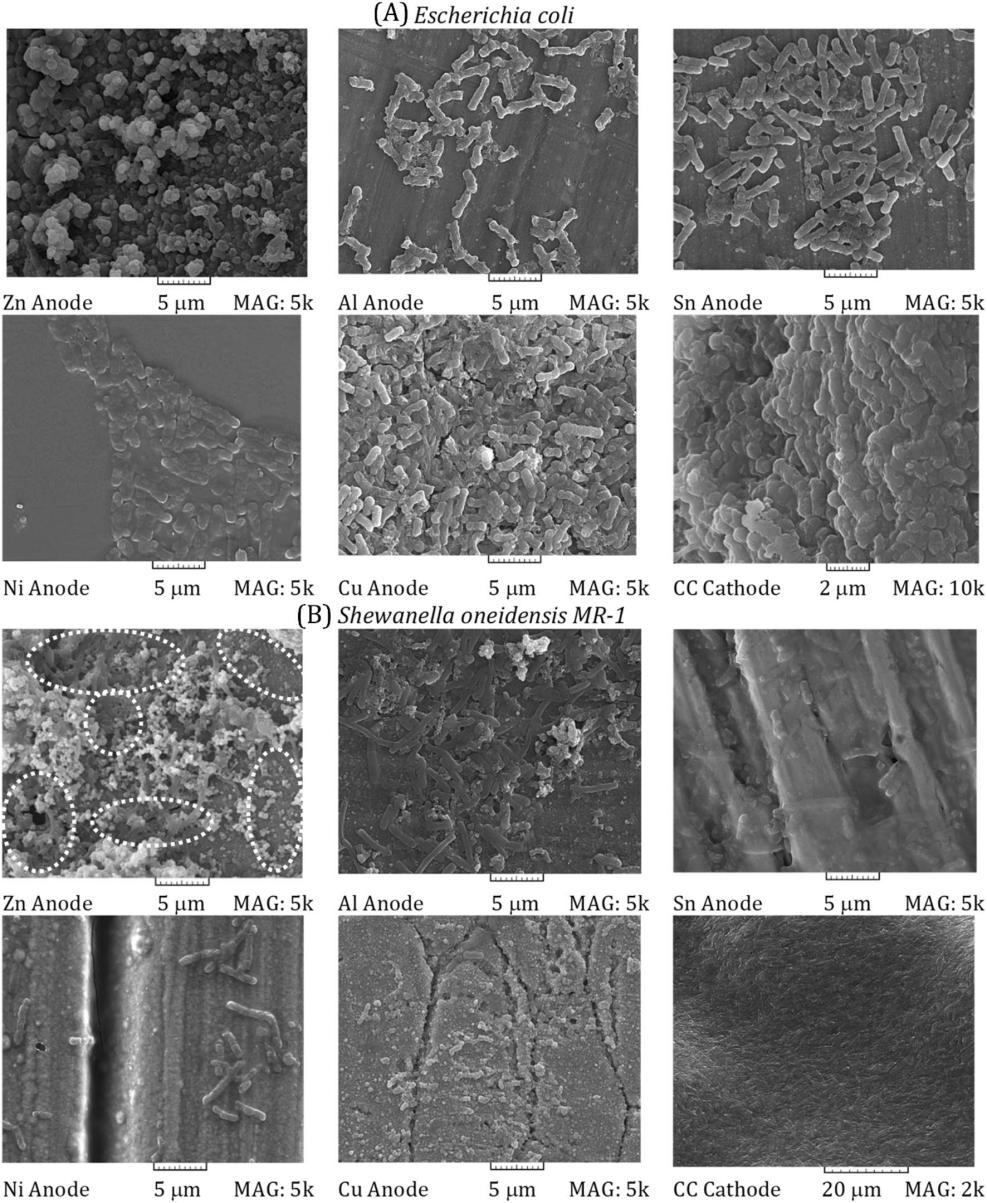
Scanning electron microscopy (SEM) images of the (**A**) *Escherichia coli* and (**B**) *Shewanella oneidensis MR-1* biofilm on the anode and cathode surface areas of the microfluidic MFCs. The related information of each SEM image was characterized.

Figure 6 shows scanning electron microscopy (SEM) images of the anodes and cathode of microfluidic MFCs inoculated with *E. coli* and S. oneidensis MR-1. The high-resolution SEM images are presented in Supplementary file. The images are ordered from the highest (i.e., Zn) to the lowest (i.e., Cu) power density. The final image of each series depicts biofilm on the carbon cloth cathode. On the cathode surface, both *S. oneidensis MR-1* and *E. coli* have formed dense biofilms. Regardless of the anode type, both species tend to adhere to the cathode surface more than they do to the anode electrodes. Ahmed and Kim demonstrated that cathodic biofilm could reduce the generated power up to 20%^56^. So, novel cathode catalysts with antibacterial activity were developed to inhibit biofilm formation on the cathode surface improved bioelectricity generation^57,58^.

As illustrated in the initial sub-figure of Fig. 6A, biofilm formation on the Zn-anode surface is uncommon. It is more accurate to state that no single bacterium or group of bacteria has a clear image. On the other hand, the naturally occurring zinc oxide nanoparticle is easily identifiable. Even though Zn-anode microfluidic MFC has the highest power density, it is evident that suspended *E. coli* may play a critical role in power generation. Between Al and Cu, the number of bacteria attached to the anode surface increased dramatically. Simultaneously, the surfaces of Al and Sn anodes are covered in sparse groups and individual bacteria, while the surfaces of Ni and Cu anodes are covered in dense groups of bacteria. Regarding the electron transfer mechanisms of *E. coli* via cytochrome c^59^, the presence of a dense biofilm may act as a barrier to delivering the mounted electron to the anode.

Along with the critical role of the standard electrode potential of the metal-based electrodes used, it appears to increase the power generation of the microfluidic MFCs inoculated with *E. coli*; it should enhance suspended bacteria growth and electron transport on the one hand while removing barriers to electron acceptors on the other hand. The role of nanoparticles in preventing the formation of a thick biofilm on the electrode surface area can be viewed as a solution, as this can be a reason to avoid biofilm formation on the Zn-anode surface area. It has been demonstrated that by coating the Ni surface of the microfluidic MFC with Ni nanoparticles, the power density can be increased by more than 30%^15^. The first possibility is that this occurs as a result of an increase in the anode surface area. However, another study found that using Zn nanoparticles on the Zn-anode surface to increase the available surface area for the *S. oneidensis MR-1* biofilm did not increase the generated power density of microfluidic MFC^60^. The presence of nanoparticles may reduce the adhesion of bacteria to the anode surface area. Future research could focus on the effect of nanoparticles on the formation of biofilms by various types of microorganisms.

To characterize the surface morphologies of the metal-based anodes, SEM images of the used anodes after removing their biofilm were captured and compared with the fresh ones (Supplementary file). Al and Ni images did not show any detectable change after ten days of operation. Local cavities can be seen partially on the Sn surface, known as pitting corrosion^61^. The natural nanostructures were present on the surface of the fresh and washed anodes in the Zn case. Besides, a few slight cracks were observed on the Zn anode after washing the biofilm, which could be considered minor corrosion. To assess the effect of anode corrosion on the performance of MFCs, Yamashita and Yokoyama examined various metals as the anode of MFCs for 350 days and reported that metal-based anodes could produce a stable current density. They also demonstrated that a specific corrosion rate could significantly decrease the generated current density^14^. In the present study, no reduction in the produced bioelectricity was observed during the operation, which indicated the negligible level and rate of corrosion.

Cu anode of the microfluidic MFC inoculated with *S. oneidensis MR-1* experienced corrosion, and a significant change in the roughness of the surface is crystal clear. On the other hand, the microfluidic MFC recruited *E. coli* as biocatalyst did not show any remarkable variation on the surface of the Cu anode. This might be related to this issue that the formed biofilm of *E. coli* on Cu anode played a role as a protective covering layer that inhibited the corrosion of the exposed surface of Cu to the anolyte of the system. On the contrary, a negligible number of bacteria can be observed on the *S. oneidensis MR-1*-inoculated anode. The corrosion of Cu anode during the operation of MFC was explained in the work of Zhu and Logan^45^. Besides, Baudler et al. demonstrated the stability of Cu as the anode of the MFC and its resistance to corrosion^13^. This could reinforce the speculation that a biofilm of a particular microorganism might play a protective role against corrosion. In the present study, *E. coli* might contribute to maintaining the Cu anode of the microfluidic MFC.

SEM images of *S. oneidensis MR-1* biofilms on microfluidic MFCs manufactured using various anodes are also shown in Fig. 6B. White dashed-line ellipses indicated the formed biofilm on the Zn-anode microfluidic MFC. The number of bacteria that formed the biofilm decreased as Cu replaced Al. In other words, Al supports a denser biofilm than Sn, and Sn supports a denser and more compact biofilm than Ni. The bacteria attached to the Cu surface are sparse and did not aggregate. The SEM images in Fig. 6 demonstrate a relationship between the abundance of formed *S. oneidensis MR-1* biofilms and power density enhancement. Microfluidic MFCs with dense biofilms achieve a higher power density than those with sparsely attached microbes on the anode.

Contrary to the microfluidic MFCs inoculated with *E. coli*, a comparison could indicate that the populated attached bacteria can be regarded as a sign of enhanced power generation in microfluidic MFCs inoculated with *S. oneidensis MR-1*. It is crystal clear that the direct electron transfer mechanism based on nanowires enables electron transport via a complex network of interconnected nanowires. That may explain why a denser and thicker *S. oneidensis MR-1* biofilm can produce a higher power density. It should be noted that future work will include SEM images of *S. oneidensis MR-1* exposed to a static magnetic field.

### Overall assessment of the microfluidic MFCs performance

**Figure 7:**
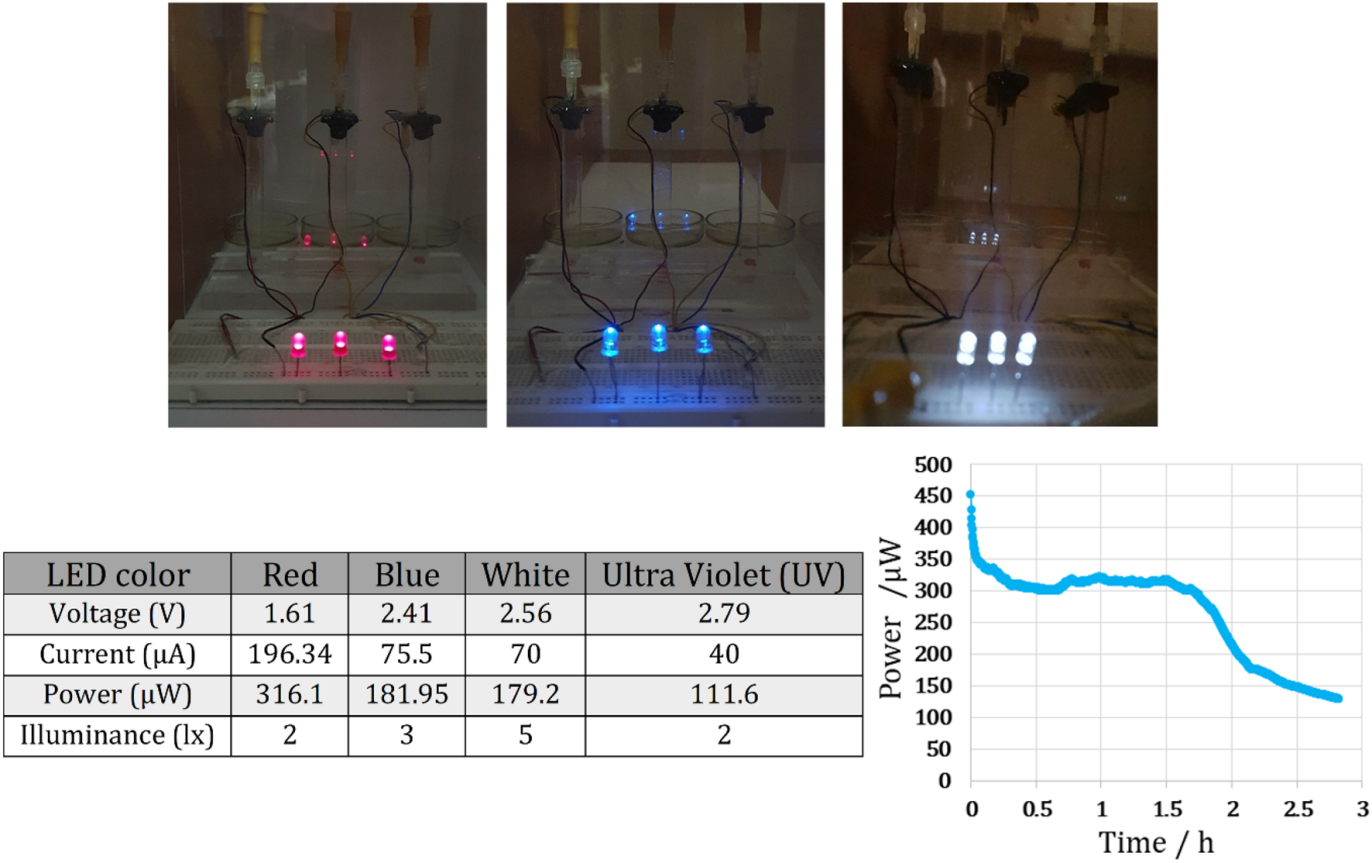
The performance of the Zn-anode microfluidic MFC inoculated with *Shewanella oneidensis MR-1* to power LEDs.

The microfluidic MFCs were evaluated overall regarding their ability to power light-emitting diodes (LEDs), and in comparison with previously published researches on microfluidic MFCs. Three Zn-anode microfluidic MFCs are connected in series to generate 4.1 V and power red, blue, white, and ultraviolet LEDs to demonstrate the capability of microfluidic MFCs to power LEDs without any external magnetic field. Figure 7 represents the illuminated LEDs, their power evolution, and the current required for each LED. Additionally, the generated light intensity was measured and reported using the light meter. The white LEDs produce significantly more light intensity, as illustrated in the figure. Since red LEDs consume more energy than other types of LEDs, the power evolution of red LEDs is depicted in Fig. 7. Despite an initial reduction in power, a consistent trend in power evolution is evident. To evaluate the sustainability of microfluidic MFCs powering red LEDs, the syringe pump was stopped, and after almost 1.75 h, the light intensity decreased from 2 to 1 lx. After 2.5 h, the LEDs were turned off. Another feature of this system is the ability to power three LEDs for more than two hours using only 150 μl of the substrate via microfluidic MFCs.

**Figure 8:**
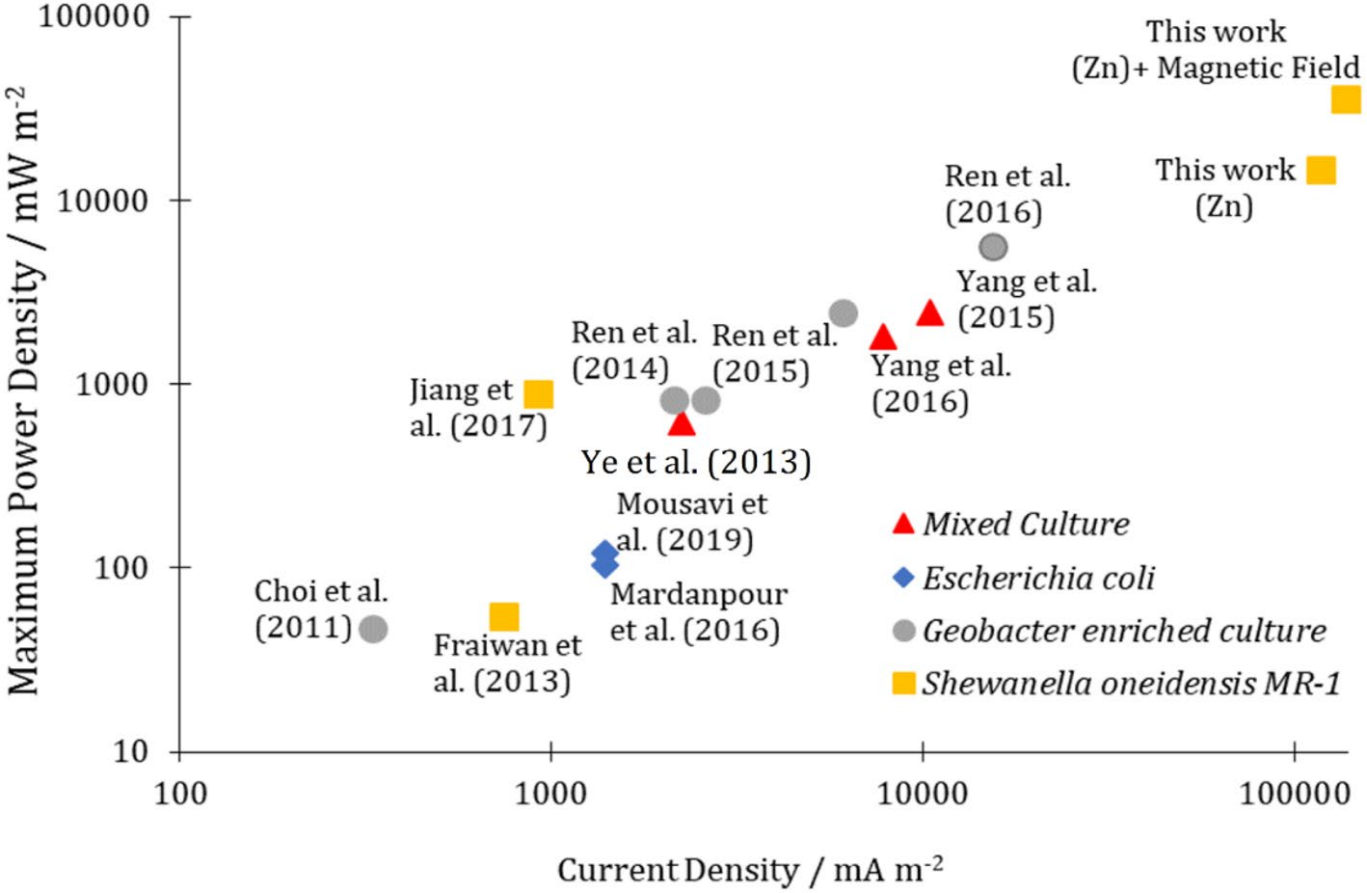
The performance of the Zn-anode microfluidic MFC inoculated with *Shewanella oneidensis MR-1* compared to previously published works.

Figure 8 shows the microfluidic MFC performance compared to the results of previously published studies. The related studies are arranged (in Table S2 of Supplementary file and Fig. 8) based on the maximum power and current densities. As can be seen, the generated power density of microfluidic MFC has been significantly improved by selecting an appropriate anode electrode, adjusting a suitable electron transfer mechanism, and applying a static magnetic field.

The manufactured microfluidic MFC does not require complex biological techniques for perfect inoculation or lengthy synthesis methods to promote electrodes. The operation was done in a conventional laboratory without the need for cleanroom conditions. The production cost of the manufactured Zn-anode microfluidic MFC inoculated with *S. oneidensis MR-1* and supported by Nd–Fe–B permanent magnets is less than $1.1. This is another exceptional feature of the system to accelerate the marketing of microfluidic MFCs for clinical and medical applications.

## Conclusion

Three simple and cost-effective methods were used to enhance bioelectricity generation of microfluidic MFCs that resulted in the highest power and current density to date. In terms of electrochemical properties, the microfluidic MFCs manufactured using metal-based electrodes exhibit the following characteristics:

- Microfluidic MFCs inoculated with *S. oneidensis MR-1* exhibit a greater power and current density than those inoculated with *E. coli*, which implies a better match between direct electron transfer mechanisms and the potential of metal-based anodes.
- *Shewanella oneidensis MR-1* requires less activation energy to extract electrons from substrate oxidation than *E. coli*, implying that metabolic pathways of *S. oneidensis MR-1* may be smoother than those of *E. coli* and less loss of nanowires compared to mobile electron shuttles.
- To enhance the power generation of the microfluidic MFCs inoculated with *E. coli*, it is necessary to reinforce the suspended bacteria growth and their electron transport on the one hand and remove the barriers to the electron acceptors on the other hand. Precipitation of nanoparticles may be critical in addressing the issue.
- In contrast to cells inoculated with *E. coli*, there is a correlation between the amount of *S. oneidensis MR-1* biofilm formed and the increase in power density. The microfluidic MFCs with dense biofilms achieve a higher power density than those with sparsely attached microbes on the anode.
- Except for the Zn-anode microfluidic MFC (2.4-fold increase), applying a static magnetic field has a detrimental effect on the power generation of the microfluidic MFCs.

Investigating the topography of biofilms using different magnetic fields and deciphering the abrupt variation that occurs by applying a static magnetic field can be future research topics.

## Supplementary Information

Supplementary Information.

## Data Availability

All data generated or analysed during this study are included in this published article.
