# Supplementary material for: Boosting microfluidic microbial fuel cells performance via investigating electron transfer mechanisms, metal-based electrodes, and magnetic field effect
Source: Sci Rep. 2022 May 6;12:7417. doi: 10.1038/s41598-022-11472-6 (PMC9076923; doi:10.1038/s41598-022-11472-6)
Supplement: Supplementary file 1 — Supplementary Information. [file 41598_2022_11472_MOESM1_ESM.docx]

**Boosting microfluidic microbial fuel cells performance via investigating electron transfer mechanisms, metal-based electrodes, and magnetic field effect**

**Mohammad Shirkosh^a^, Yousef Hojjat^*a^, Mohammad Mahdi Mardanpour^b^**

**^a^** Department of Mechanical Engineering, Tarbiat Modares University, Tehran, Iran.

Email: yhojjat@modares.ac.ir

^b^ Department of Bioengineering, McGill University, Montreal, Quebec, Canada.

**The power density curves of the microfluidic MFCs engaged with aluminum and nickel anodes**

The Al-anode microfluidic MFC, inoculated with *Shewanella oneidensis MR-1*, produced the maximum current density of 43600 mA m^-2^ obtained in Fig. S1D was more than 2.8-fold that of the highest value in the previous study (i.e., 15510 mA m^-2^)^1^. Ni (Fig. S1A) demonstrated superior performance (approximately 28% higher power density than Cu) as the anode of the microfluidic MFC when *Escherichia coli* was used as the catalyst, implying a more biocompatible surface for the Ni than the Cu provided surface. Furthermore, Ni can provide a higher power density over a broader range of substrate flow rates. The results obtained using a microfluidic MFC inoculated with *Escherichia coli* are consistent with those previously published^2^. While Cu did not generate significantly more power in terms of electron transfer mechanisms (i.e., for both *Escherichia coli* and *Shewanella oneidensis MR-1*), the microfluidic MFC with Ni (Fig. S1C) as the anode and inoculated with *Shewanella oneidensis MR-1* generated more power (by more than 2-fold) than the cell inoculated with *Escherichia coli*. The contribution of nanowires to the enhancement of the power generation of Ni-anode microfluidic MFC is undeniable.

**
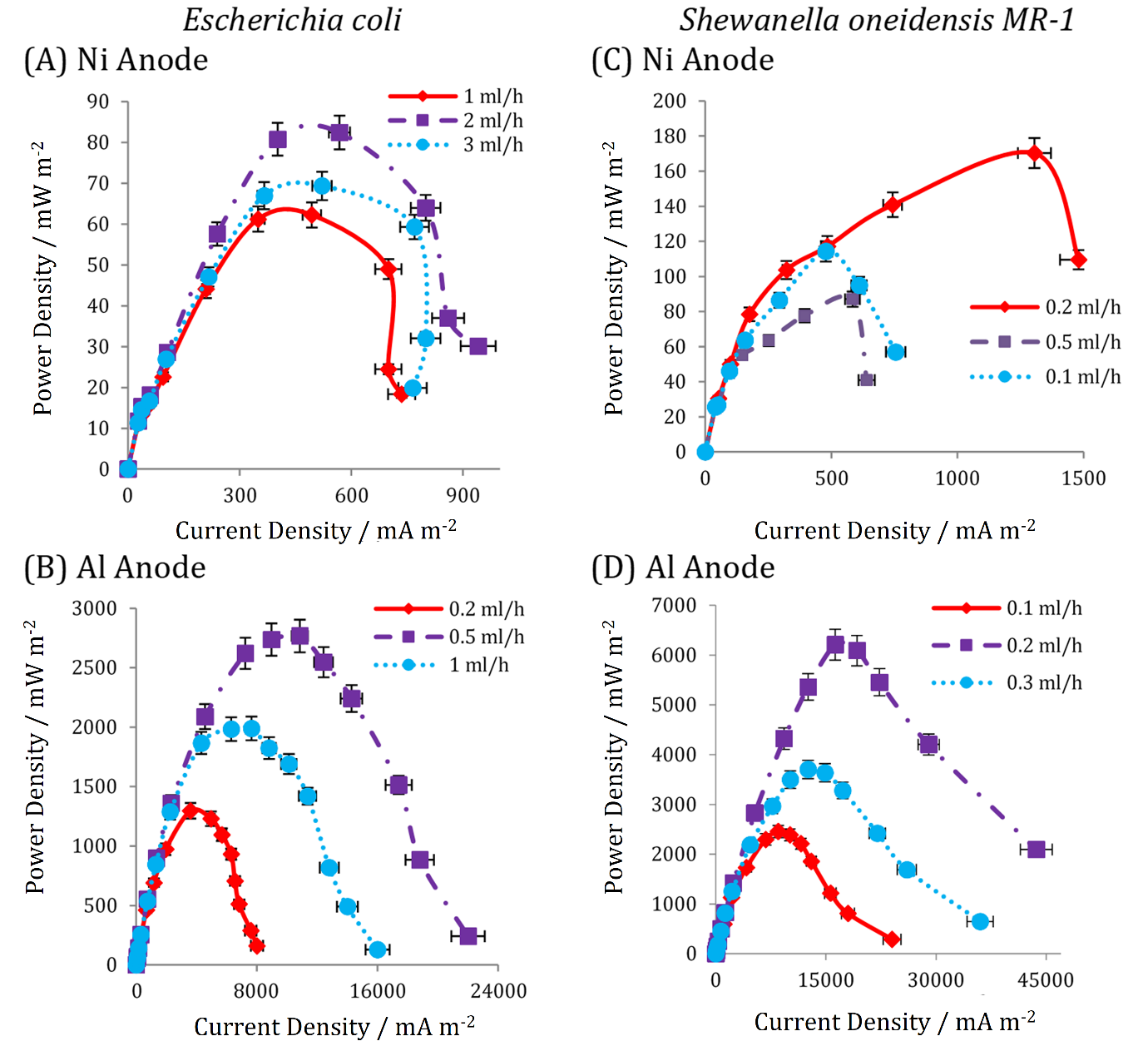
**

Fig. S1. The power density curves for the microfluidic MFCs with different anode electrodes engaged with *Escherichia coli* ((A) Ni and (B) Al) and *Shewanella oneidensis MR-1* ((C) Ni and (D) Al) under different substrate injection rates. The error bars represent the variation of power and current densities among repeated experiments.

Table S1. The summary of the electrochemical analysis results of the microfluidic MFCs.

| Biocatalyst | Anode | Internal Resistance (kΩ) | Opt. Flow rate / ml h^-1^ | Maximum Current Density /  mA m^-2^ | Maximum Power Density /  mW m^-2^ | Maximum Power Density under the static Magnetic Field /  mW m^-2^ |
| --- | --- | --- | --- | --- | --- | --- |
| *Escherichia coli* | Zn | 0.22 | 1 | 76000 | 7281 | - |
|  | Al | 0.47 | 0.5 | 22000 | 2767 | - |
|  | Sn | 2 | 0.3 | 4600 | 380 | - |
|  | Ni | 5.1 | 2 | 941 | 82 | - |
|  | Cu | 5.1 | 0.1 | 809 | 64 | - |
| *Shewanella oneidensis MR-1* | Zn | 0.05 | 0.3 | 118000 | 14592 | 35295 |
|  | Al | 0.47 | 0.2 | 43636 | 6209 | 2073 |
|  | Sn | 0.68 | 0.2 | 9000 | 781 | 681 |
|  | Ni | 2 | 0.2 | 1480 | 170 | 124 |
|  | Cu | 2 | 1 | 840 | 58 | 45 |

Table S2. The performance comparison between the current microfluidic MFCs and previously reported microfluidic MFCs.

| Anode | Substrate | Anolyte volume  (µl) | Biocatalyst | Power density (W m^-3^) | Current density (A m^-3^) | Internal resistance (kΩ) | Reference |
| --- | --- | --- | --- | --- | --- | --- | --- |
| Zinc in a static magnetic field (86 mT) | TSB* | 50 | *S. oneidensis MR-1* | 35294 | 138181 | 0.05 | This study |
| Zinc | TSB | 50 | *S. oneidensis MR-1* | 14592 | 118000 | 0.05 | This study |
| 3D graphene | Acetate | 50 | *Geobacter* enriched | 11220 | 31020 | 0.219 | ^1^ |
| Graphene oxide on nickel foam | Acetate | 50 | Mixed culture | 1181.4 | 4000 | 0.7 | ^3^ |
| 3D graphene foam | TSB | 58.8 | *S. oneidensis MR-1* | 745 | NA | 7.3 | ^4^ |
| Nickel | NB** | 50 | *E. coli* | 104 | 1400 | 10 | ^2^ |

* Tryptic soy broth (TSB)

** Nutrient Broth (NB)

**The high-resolution Scanning electron microscopy (SEM) images.**

| **(A) Zn Anode** | **(B) Zn Anode** |
| --- | --- |
| 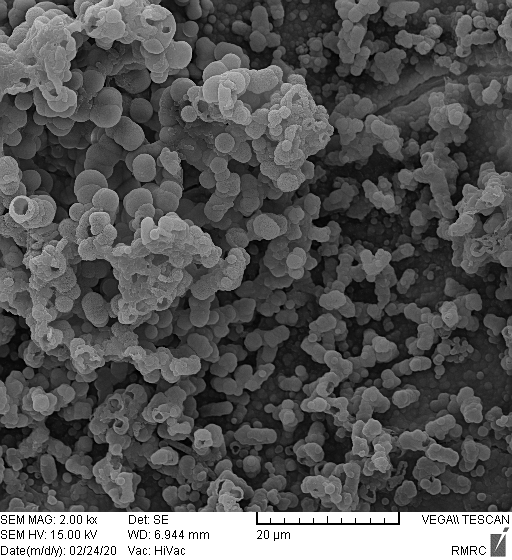 | 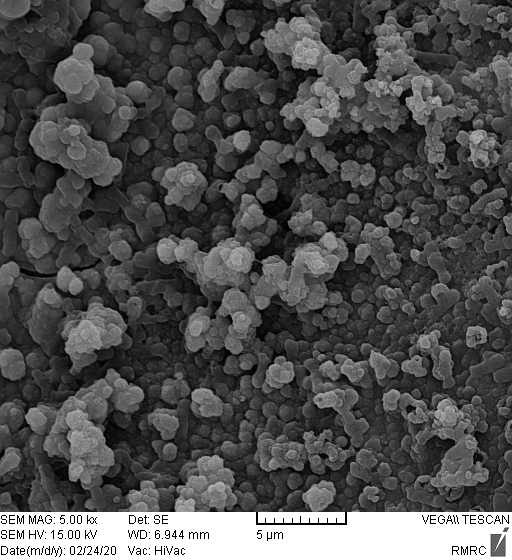 |
| **(C) Carbon cloth Cathode** |  |
| 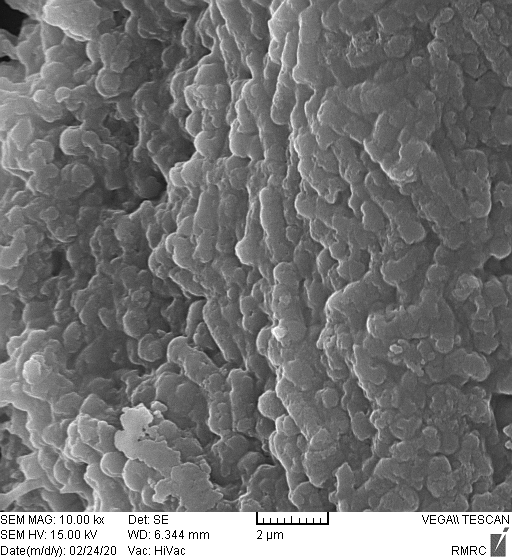 |  |

Fig. S2. Scanning electron microscopy (SEM) images of the *Escherichia coli* biofilm on the surface areas of Zn anode with (A) 2000 and (B) 5000 magnification, and (C) carbon cloth cathode with 10000 magnification.

| **(A) Al Anode** | **(B) Al Anode** |
| --- | --- |
| 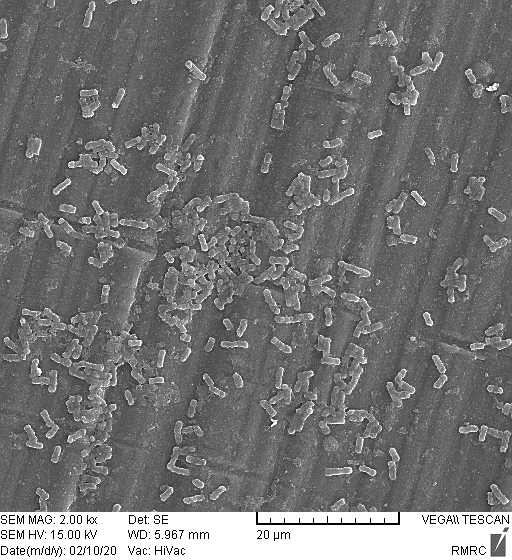 | 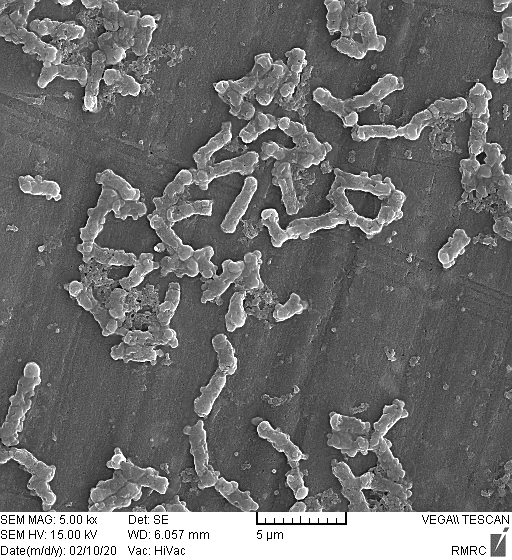 |
| **(C) Carbon cloth Cathode** |  |
| 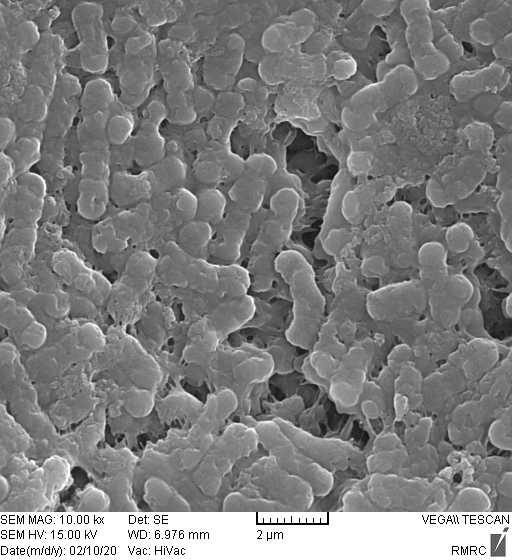 |  |

Fig. S3. Scanning electron microscopy (SEM) images of the *Escherichia coli* biofilm on the surface areas of Al anode with (A) 2000 and (B) 5000 magnification, and (C) carbon cloth cathode with 10000 magnification.

| **(A) Sn Anode** | **(B) Sn Anode** |
| --- | --- |
| 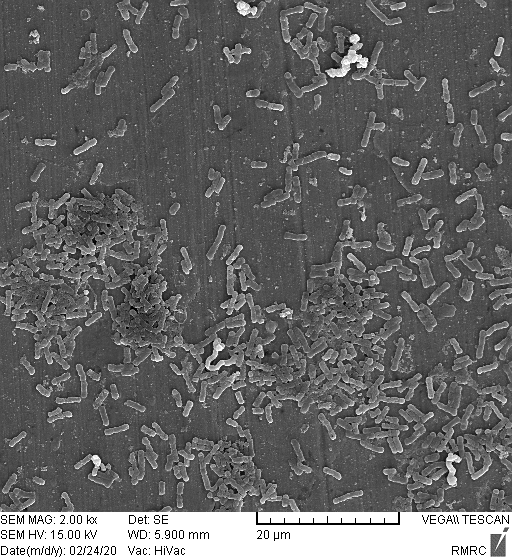 | 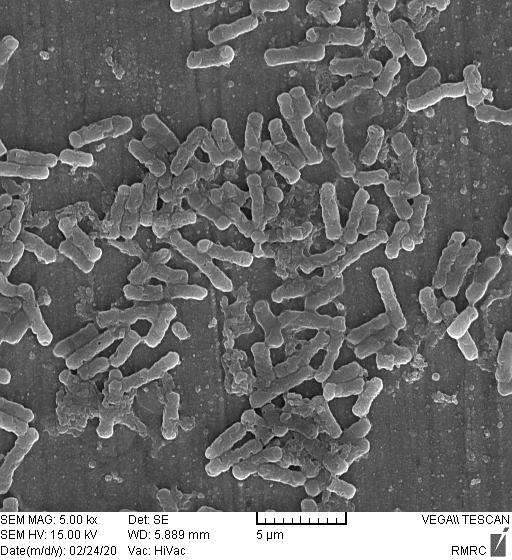 |
| **(C) Carbon cloth Cathode** |  |
| 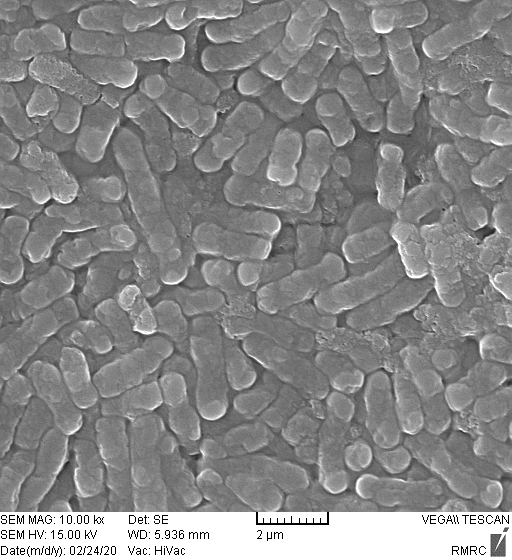 |  |

Fig. S4. Scanning electron microscopy (SEM) images of the *Escherichia coli* biofilm on the surface areas of Sn anode with (A) 2000 and (B) 5000 magnification, and (C) carbon cloth cathode with 10000 magnification.

| **(A) Ni Anode** | **(B) Ni Anode** |
| --- | --- |
| 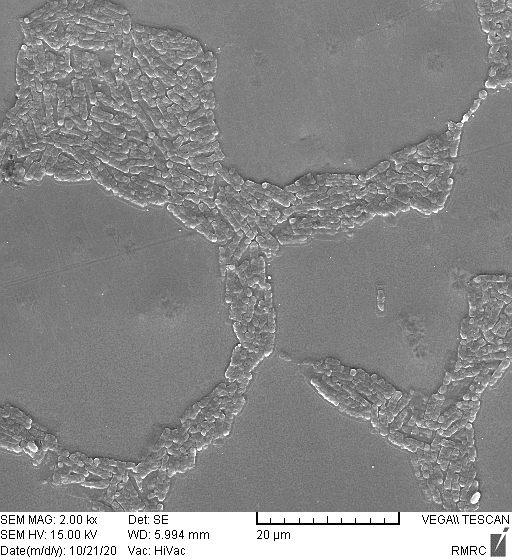 | 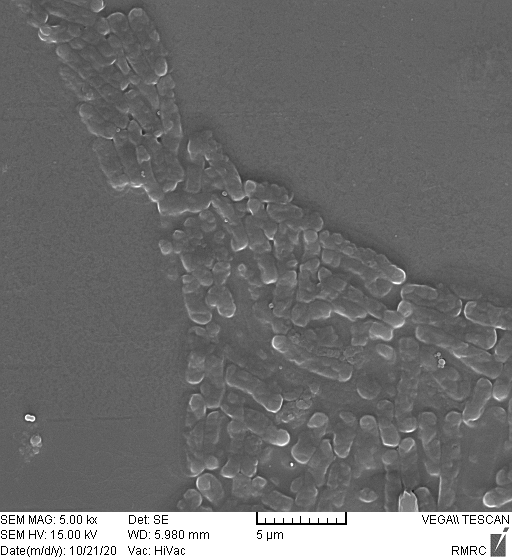 |
| **(C) Carbon cloth Cathode** |  |
| 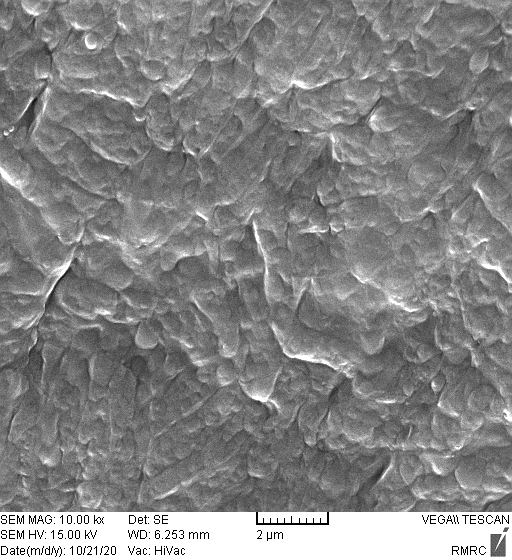 |  |

Fig. S5. Scanning electron microscopy (SEM) images of the *Escherichia coli* biofilm on the surface areas of Ni anode with (A) 2000 and (B) 5000 magnification, and (C) carbon cloth cathode with 10000 magnification.

| **(A) Cu Anode** | **(B) Cu Anode** |
| --- | --- |
| 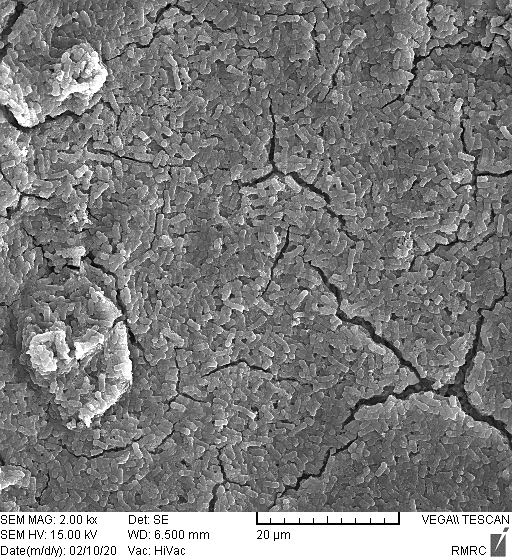 | 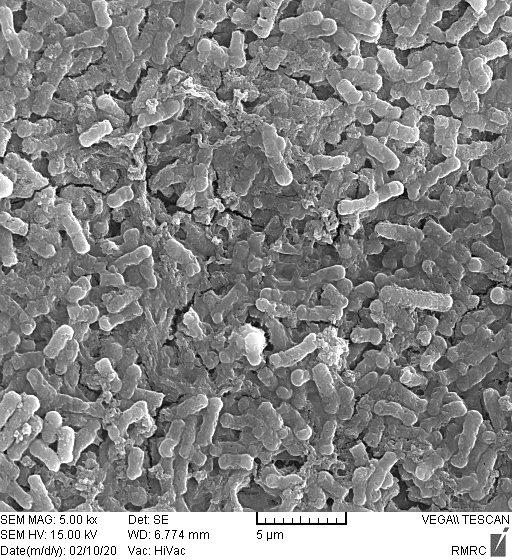 |
| **(C) Carbon cloth Cathode** |  |
| 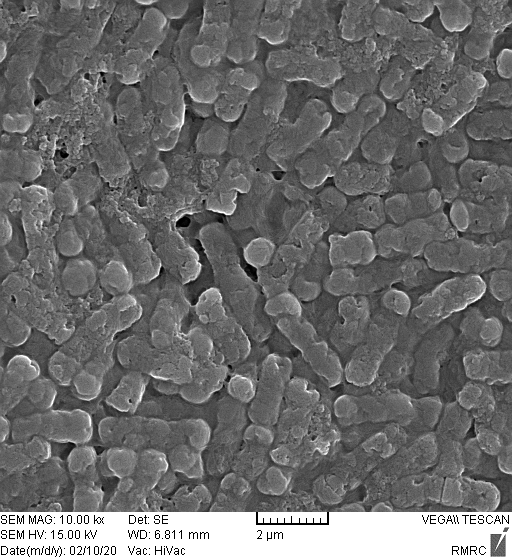 |  |

Fig. S6. Scanning electron microscopy (SEM) images of the *Escherichia coli* biofilm on the surface areas of Cu anode with (A) 2000 and (B) 5000 magnification, and (C) carbon cloth cathode with 10000 magnification.

| **(A) Zn Anode** | **(B) Zn Anode** |
| --- | --- |
| 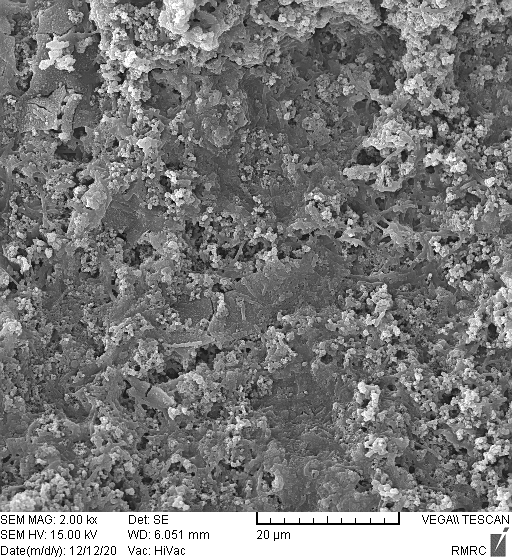 | 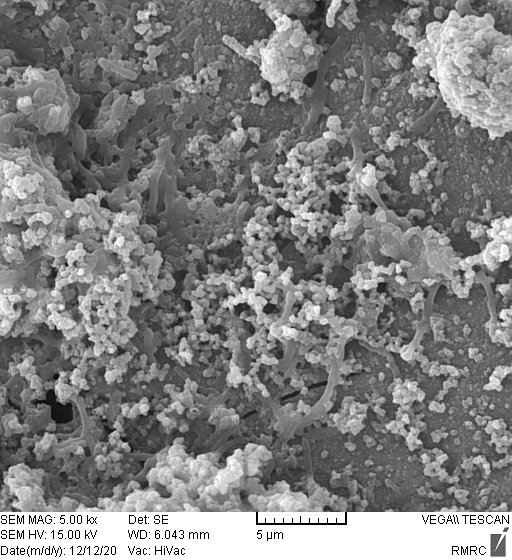 |
| **(C) Carbon cloth Cathode** |  |
| 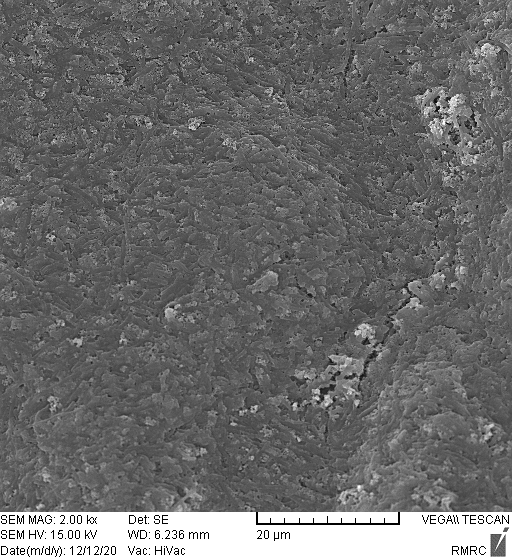 |  |

Fig. S7. Scanning electron microscopy (SEM) images of the *Shewanella oneidensis MR-1* biofilm on the surface areas of Zn anode with (A) 2000 and 5000 magnification, and (C) carbon cloth cathode with 2000 magnification.

| **(A) Al Anode** | **(B) Al Anode** |
| --- | --- |
| 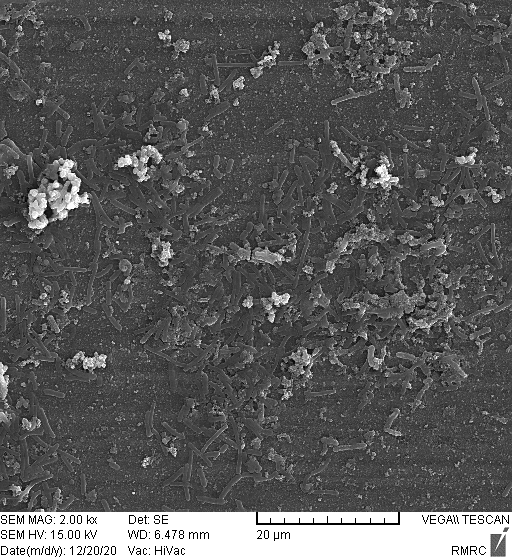 | 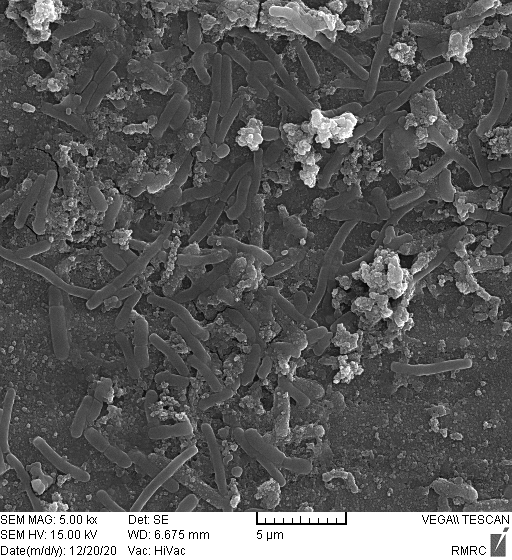 |
| **(C) Carbon cloth Cathode** |  |
| 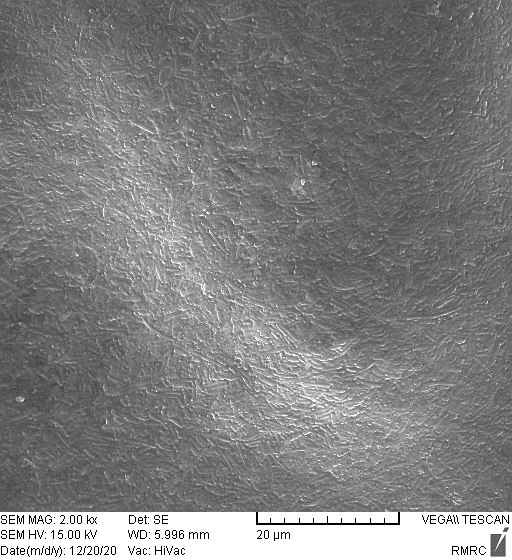 |  |

Fig. S8. Scanning electron microscopy (SEM) images of the *Shewanella oneidensis MR-1* biofilm on the surface areas of Al anode with (A) 2000 and (B) 5000 magnification, and (C) carbon cloth cathode with 2000 magnification.

| **(A) Sn Anode** | **(B) Sn Anode** |
| --- | --- |
| 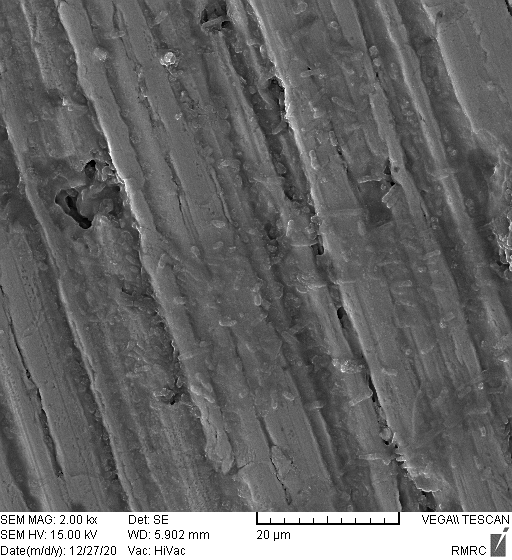 | 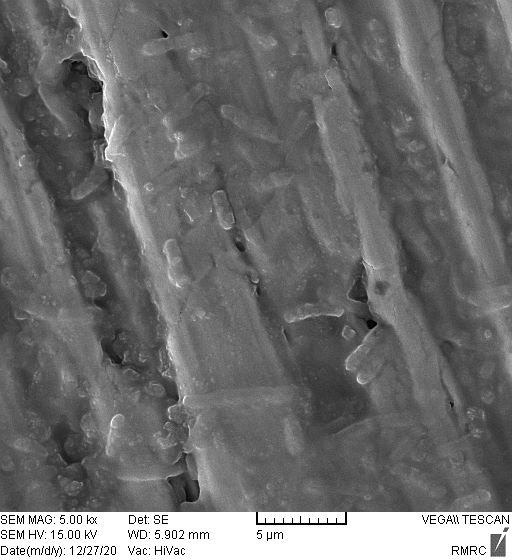 |
| **(C) Carbon cloth Cathode** |  |
| 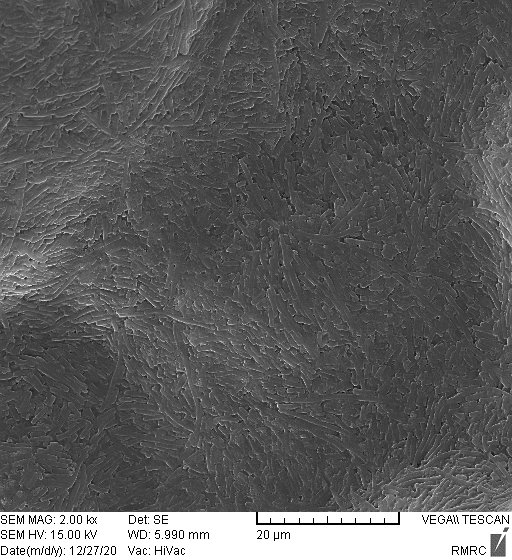 |  |

Fig. S9. Scanning electron microscopy (SEM) images of the *Shewanella oneidensis MR-1* biofilm on the surface areas of Sn anode with (A) 2000 and (B) 5000 magnification, and (C) carbon cloth cathode with 2000 magnification.

| **(A) Ni Anode** | **(B) Ni Anode** |
| --- | --- |
| 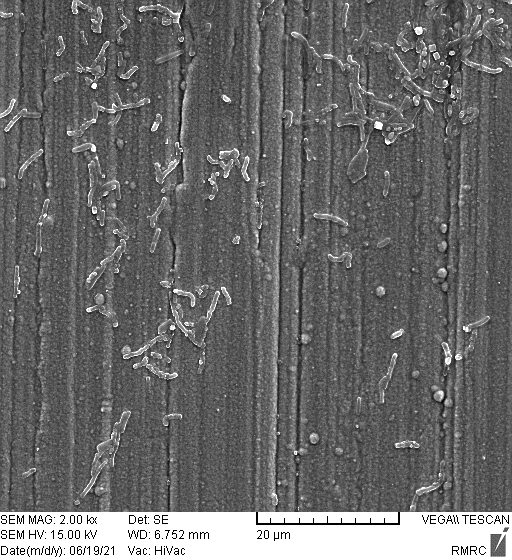 | 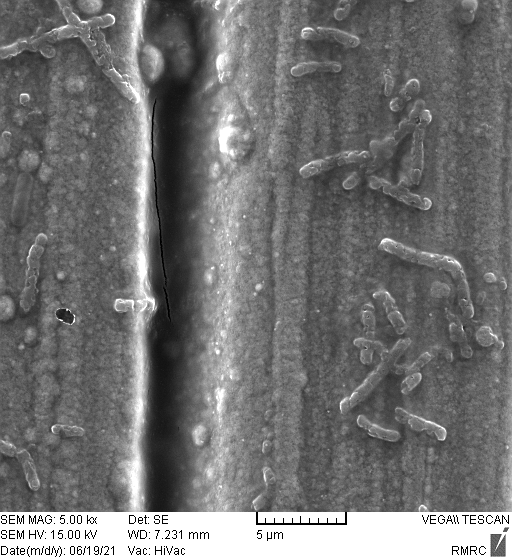 |
| **(C) Carbon cloth Cathode** |  |
| 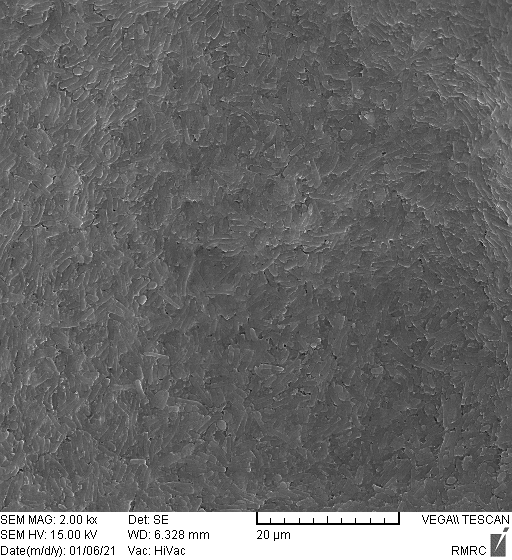 |  |

Fig. S10. Scanning electron microscopy (SEM) images of the *Shewanella oneidensis MR-1* biofilm on the surface areas of Ni anode with (A) 2000 and (B) 5000 magnification, and (C) carbon cloth cathode with 2000 magnification.

| **(A) Cu Anode** | **(B) Cu Anode** |
| --- | --- |
| 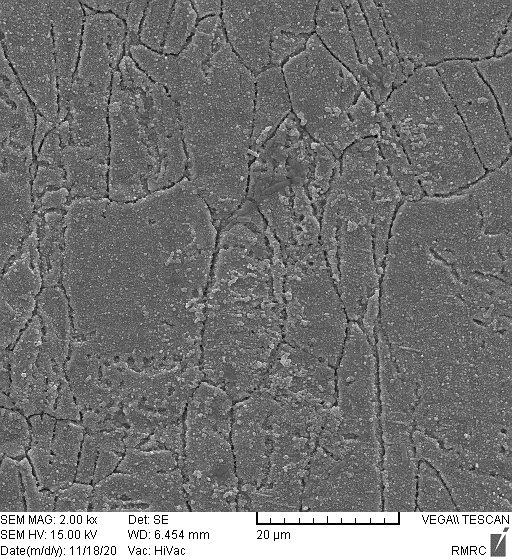 | 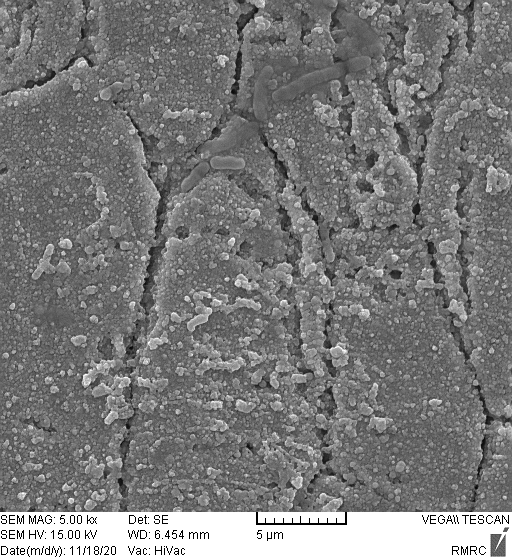 |
| **(C) Carbon cloth Cathode** |  |
| 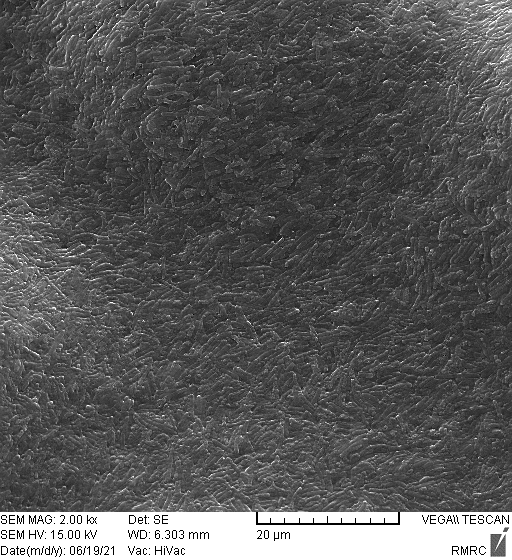 |  |

Fig. S11. Scanning electron microscopy (SEM) images of the *Shewanella oneidensis MR-1* biofilm on the surface areas of Cu anode with (A) 2000 and (B) 5000 magnification, and (C) carbon cloth cathode with 2000 magnification.


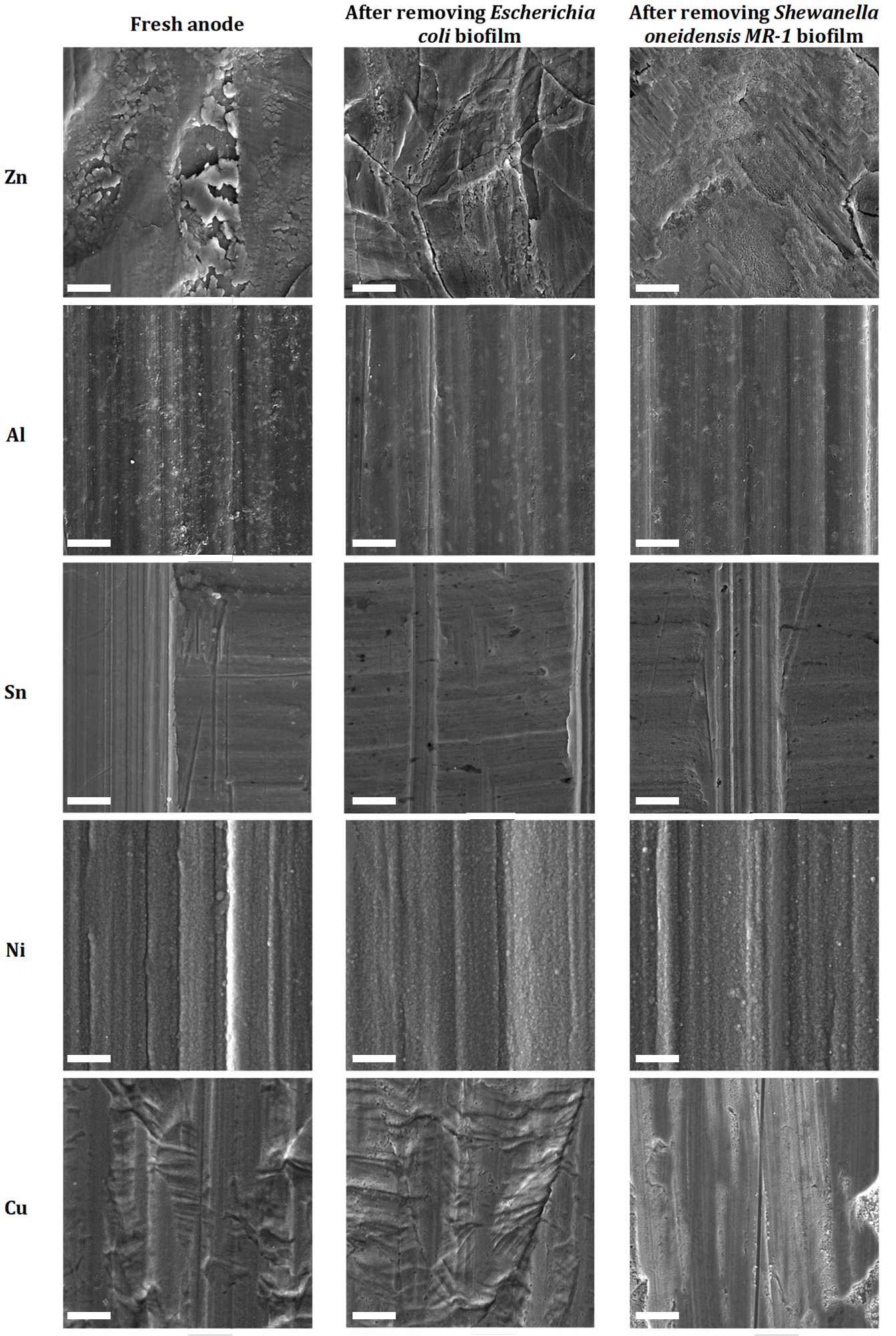


Fig. S12. Scanning electron microscopy (SEM) images of metal-based anodes in fresh state and after removing *Escherichia coli* and *Shewanella oneidensis MR-1* biofilm with 5000 magnification (scale bars represent 5µm).

**References**

1 Ren, H., Tian, H., Gardner, C. L., Ren, T.-L. & Chae, J. A miniaturized microbial fuel cell with three-dimensional graphene macroporous scaffold anode demonstrating a record power density of over 10000 W m− 3. *Nanoscale* **8**, 3539-3547 (2016).

2 Mardanpour, M. M. & Yaghmaei, S. Characterization of a microfluidic microbial fuel cell as a power generator based on a nickel electrode. *Biosensors and Bioelectronics* **79**, 327-333 (2016).

3 Yang, Y. *et al.* A three-dimensional nitrogen-doped graphene aerogel-activated carbon composite catalyst that enables low-cost microfluidic microbial fuel cells with superior performance. *Journal of Materials Chemistry A* **4**, 15913-15919 (2016).

4 Jiang, H., Ali, M. A., Xu, Z., Halverson, L. J. & Dong, L. Integrated microfluidic flow-through microbial fuel cells. *Scientific reports* **7**, 1-12 (2017).
